# A Review of the Potential Benefits of Herbal Medicines, Small Molecules of Natural Sources, and Supplements for Health Promotion in Lupus Conditions

**DOI:** 10.3390/life13071589

**Published:** 2023-07-19

**Authors:** Ardalan Pasdaran, Bahareh Hassani, Ali Tavakoli, Ekaterina Kozuharova, Azadeh Hamedi

**Affiliations:** 1 Department of Pharmacognosy, School of Pharmacy, Shiraz University of Medical Sciences, Shiraz 7146864685, Iran; pasdaran@sums.ac.ir; 2 Medicinal Plants Processing Research Center, Shiraz University of Medical Sciences, Shiraz 7146864685, Iran; 3 Student Research Committee, School of Pharmacy, Shiraz University of Medical Sciences, Shiraz 7146864685, Iran; hasanibahareh@yahoo.com; 4 Research Center for Traditional Medicine and History of Medicine, Department of Persian Medicine, School of Medicine, Shiraz University of Medical Sciences, Shiraz 7134845794, Iran; tavakkolia@sums.ac.ir; 5 Department of Pharmacognosy, Faculty of Pharmacy, Medical University of Sofia, 1431 Sofia, Bulgaria; ina_kozuharova@yahoo.co.uk

**Keywords:** systemic lupus erythematosus, clinical, autoimmune diseases, natural product, supplement, medicinal plant, inflammation, immunomodulatory, review, herbal medicine

## Abstract

The Latin word lupus, meaning wolf, was in the medical literature prior to the 1200s to describe skin lesions that devour flesh, and the resources available to physicians to help people were limited. The present text reviews the ethnobotanical and pharmacological aspects of medicinal plants and purified molecules from natural sources with efficacy against lupus conditions. Among these molecules are artemisinin and its derivatives, antroquinonol, baicalin, curcumin, emodin, mangiferin, salvianolic acid A, triptolide, the total glycosides of paeony (TGP), and other supplements such as fatty acids and vitamins. In addition, medicinal plants, herbal remedies, mushrooms, and fungi that have been investigated for their effects on different lupus conditions through clinical trials, in vivo, in vitro, or in silico studies are reviewed. A special emphasis was placed on clinical trials, active phytochemicals, and their mechanisms of action. This review can be helpful for researchers in designing new goal-oriented studies. It can also help practitioners gain insight into recent updates on supplements that might help patients suffering from lupus conditions.

## 1. Introduction

Systemic lupus erythematosus (SLE) is an autoimmune disease involving multiple organs and clinical manifestations. SLE is more common in young women. The incidence of SLE in males compared to females is 1:5~10 [1,2]. In SLE, autoantibodies and antibody-immune complexes are produced that eventually cause damage to body tissues and induce inflammation [3,4]. SLE patients experience relapsing and remission courses [5]. In SLE, various organs can be involved, including the skin, kidneys, joints, heart, lungs, liver, and blood vessels [6,7]. Since different organs are involved in SLE, a variety of indices can be used to assess the status of diseases, such as the Systemic Lupus Erythematosus Disease Activity Index (SLEDAI), Safety of Estrogens in Lupus Erythematosus National Assessment (SELENA), or British Isles Lupus Activity Group (BILAG) index [8].

The level of anti-double-stranded DNA (anti-dsDNA) antibodies is associated with disease activity, and anti-dsDNA plays an important role in the pathogenesis of SLE. In some cases, the goal of treatment is to bring the level of anti-dsDNA antibodies back to normal (5). Mechanisms involved in kidney damage due to lupus nephritis include dysregulation of T-regulatory cells due to overactivity of B and T lymphocytes, activation of inflammatory responses, improper production of autoantibodies, and deposition of immune complexes in kidney tissue [9,10].

The current medications used to treat SLE include glucocorticoids, immunosuppressive drugs, non-steroidal anti-inflammatory drugs, anti-malarial drugs, systemic lymph node irradiation therapy, and plasma treatment. Despite this, the morbidity and mortality ratios in SLE patients are still unacceptably high [6,11]. The mentioned medications lead patients to be exposed to side effects and also reduce the patient’s quality of life [12]. This encourages patients to try complementary and alternative medicines such as herbal remedies, medicinal plants, phytochemicals, vitamins and mineral supplements, acupuncture, moxibustion, and spiritual therapy such as yoga. Moreover, tremendous efforts have been made by researchers to develop safe and efficient drugs and supplements from natural molecules and their synthetic derivatives for the condition.

Some previous articles can be found on the treatment of lupus conditions that are not on natural products or supplements and mostly discussed orthodox medicine [13,14,15,16,17,18]. Some other review articles discussing natural products on a specific condition of lupus, such as cutaneous lupus [19] or lupus nephritis [20], can be found in the literature. Some discussed only a specific natural compound such as curcumin [21], omega-3 fatty acids [22], and triptolide [23]. On the other hand, some other review articles reflected a specific traditional medicine approach such as traditional Chinese medicine (TCM), traditional Iranian medicine (TIM) [24], or Ayurveda [25], which mostly focused on polyherbal formulations and plant extracts that are composed of a complex mixture of phytochemicals with a holistic approach. Some of these articles have neglected minerals, vitamins, and pure active phytoconstituents. Conversely, some reviews and meta-analyses specifically focused on fatty acids [22,26,27] or vitamins [28,29]. Moreover, some articles focused mostly on pharmacology and possible mechanisms of action of a group of natural components without scoping clinical trials or meta-analyses [30]. All the mentioned articles provided precious information for specific groups of researchers and highlighted specific aspects of treatment and drug development for lupus conditions. But a comprehensive systematic review that simultaneously gives detailed evidence-based information on the efficacy of natural products and supplements on different lupus conditions, which helps practitioners and clinicians to understand the mechanism of action and the level of evidence for any of these molecules, herbs, or supplements and researchers to design new goal-oriented experimental studies or clinical trials, seems to be lacking. The present article tries to cover the last-mentioned points in this paragraph and reviewed natural molecules, phytochemicals, medicinal plants, fungi, vitamins, minerals, and other supplements that have been reported to be beneficial for lupus conditions, with special emphasis on clinical trials and the molecules’ mechanism of actions, as well as their adverse effects and toxicity.

## 2. Materials and Methods

A comprehensive literature search was conducted in PubMed, Cochrane Library, Web of Science, Scopus, the National Library of Medicine (NLM) catalog, and Google Scholar, from January 1970 up until January 2023. The obtained records were assessed for eligibility in accordance with the PRISMA 2000 guidelines.

**Inclusion criteria:** Combinations of various keywords including systemic lupus erythematosus, SLE, lupus nephritis, or lupus AND natural molecule, natural medicine, phytochemicals, herbal medicine, medicinal plants, fungi, mushrooms, minerals, vitamins, fatty acids, supplements, nutrition, meta-analysis, and clinical trial, toxicity, and side effects have been considered in the search strategy. Moreover, the compound names and scientific names of the identified plants were searched again with keywords related to lupus. No restriction was set on the language. A special focus was set on reported purified molecules from natural sources including artemisinin and its derivatives, antroquinonol, baicalin, curcumin, emodin, mangiferin, salvianolic acid A, triptolide, the total glycosides of paeony (TGP), some fatty acids, and vitamins.

**Exclusion criteria:** (1) Duplicate article; (2) addressed a natural compound, but not related to lupus conditions; (3) article on lupus conditions, but not related to natural compounds; (4) did not address specific natural compounds or plant extracts; (5) studies involving entirely synthetic molecules or antibodies; (6) herbal remedies from traditional medicine that had not been scientifically evaluated for lupus conditions; and (7) polyherbal formulations from traditional medicine.

**Extraction of data:** The following entries were included: (1) study ID; (2) title; (3) aim or objective of the study; (4) study design; (5) possible conflicts of interests for study authors; (6) participants/population description (humans, animal, cell lines, etc.); (7) the total number of participants; (8) inclusion criteria; (9) exclusion criteria; (10) total number of experimental repeats; (11) the tested material (plants/compounds/extracts, fungi, mushroom, pure molecule, etc.); (12) the active ingredients or molecule were tested; (13) the used positive or negative controls and placebo; (14) primary outcome and findings on lupus conditions (lupus nephritis, cutaneous lupus, systemic lupus erythematosus (SLE), etc.)—was the intervention statically effective? (15) primary outcome—inflammation—was the intervention statically effective? (16) primary outcome—immunomodulation—was the intervention statically effective? (17) primary outcome—other organ systems—was the intervention statically efficacious? (18) the reported adverse effects for molecules or extracts; (19) the reported interaction (with drugs, food, or herbs) for the tested molecules or extracts.

## 3. Results

The search strategy yielded 14,300 studies. The titles or abstracts were reviewed to exclude duplicates or irrelevant ones. Excluded were 13,887 records that were identified as irrelevant, duplicate, or not reliable. As a result, 413 studies were included in the review and 74 were included in the synthesized tables.

### 3.1. Ethnobotany

The Latin word lupus, meaning wolf, was in the medical literature prior to the 1200s to describe skin lesions that devour flesh, and the resources available to physicians to help people were limited [31,32]. Traditional knowledge on how to deal with this condition involves the use of several medicinal plants or plant-based mixtures. Ethnobotanical and ethnopharmacological studies reveal that *Cinchona* spp. [33] and “Thanatka” made of *Hesperethusa crenulata* and *Limonia acidissima* bark [34] have dermatologic uses, specifically in the treatment of lupus erythematosus. Also, sieketroos *Arctopus* species [35], *Juniperus* species [36], *Onopordum acanthium* [37], and *Centella asiatica* [38] were documented to treat systemic lupus erythematosus. According to Iranian traditional medicine (traditional Persian medicine), infectious diseases and fever are the main reasons for nephritis, which is called “Varam-e-Kolye”. Several medicinal plants have been advised to control for lupus nephritis or “Varam-e-Kolye”, which are *Anethum graveolens* L., *Carum carvi* L., *Coriandrum sativum* L., *Cucurbita pepo* L., *Cydonia oblonga* Mill., *Ficus carica* L., *Linum usitatissimum* L., *Melissa officinalis* L., *Prunus amygdalus*, and *Ziziphus jujuba* Mill. Some recent research reported nephroprotective and anti-inflammatory properties of these plants [24,39,40].

As examples, *Cuminum cyminum* L. (in Persian كرويا or زيره سبز), *Carum carvi* L. (in Persian كمون كرماني or زيره سياه), *Lagoecia cuminoides* L. (in Persian زيره وحشي or قردمانا) [41,42], and *Bunium persicum* (Boiss.) Fedtch (in Persian Zire Kermani) [39] are other plants advised for Varam-e-Kolye and/or other kidney diseases such as “Riah-e-Gorde” [41,42]. Carvia (كرويا) is the Arabic version of the Latin word “craviya” or the Syriac word “Ceravi”; in Greek the word is “Azhamyon”, in Roman “Fadroni”, and in Arabic “Taghdeh”, “Taghrad”, and “Comone Roomi” [42]. *B. persicum* has shown antiglycation, antioxidant, anti-inflammatory, and nephroprotective (possibly due to antiglycation) effects [43,44,45].

According to the literature, several medicinal plants and fungi have been considered to be beneficial for conditions related to lupus. This review focused on those that are evidence-based with in vitro or in vivo studies or clinical trials. The ethnobotanical aspects of these plants are summarized in Table 1.

### 3.2. Purified Molecules from Natural Sources

**Figure 1:**
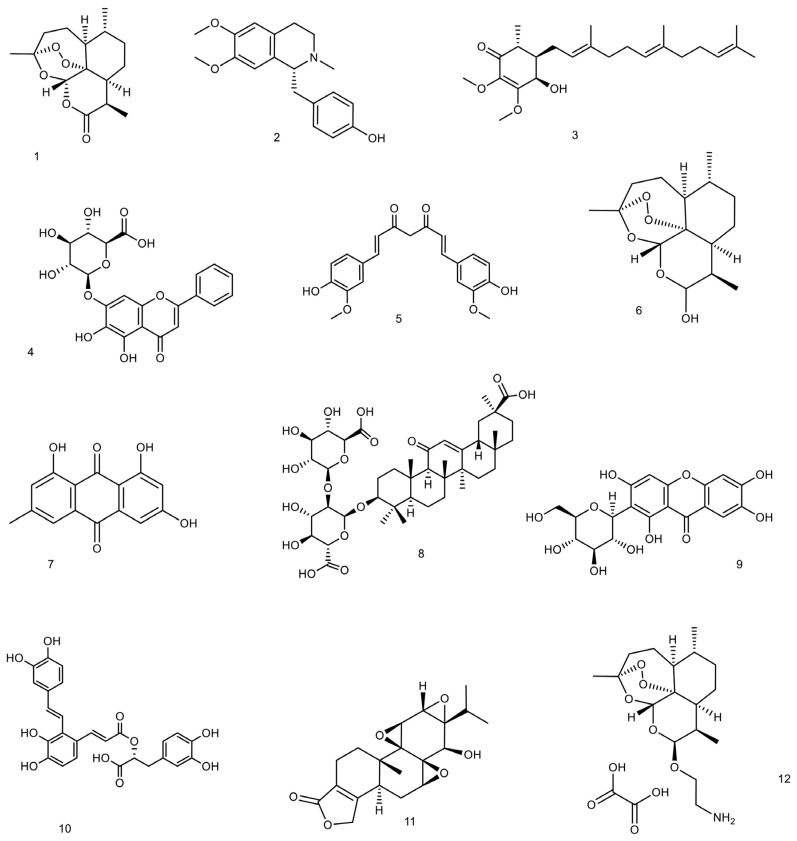
The structures of small molecules from natural sources and their derivatives with reported efficacy against lupus conditions. The structures are 1: (+)—artemisinin, 2: (S)—armepavine, 3: antroquinonol, 4: baicalin, 5: curcumin, 6: dihydroartemisinin, 7: emodin, 8: glycyrrhizic acid, 9: mangiferin, 10: salvianolic acid A, 11: triptolide, and 12: β-aminoarteether maleate.

Different herbal remedies, medicinal plants, and mushrooms have been utilized to cure a range of medical ailments in both developing and developed communities. Additionally, it is estimated that roughly 25% of currently marketed medicines were developed from the primary or secondary metabolites of natural medicines [90]. On the other hand, the absence of a well-organized regulatory and legal framework for herbal products has caused the World Health Organization (WHO) to express worry regarding the efficacy and safety of herbal treatments [91]. Due to varying growth circumstances and harvesting times, different primary and secondary metabolites have varying concentrations in medicinal plants [92]. These problems motivate researchers to find and purify the medicinal plant’s active components. Researchers have gained a greater understanding of the mechanisms of action by working with highly purified compounds. When compared to herbal extracts, pure natural molecules are more reliable at determining dosage and detecting unwanted effects or potential toxicities. Moreover, natural molecules can be considered lead compounds for developing new drugs. In the case of lupus, several natural products and their derivatives, in purified and structure-elucidated form, have been reported to exhibit considerable therapeutic potential. Although the mechanisms of action of some of these molecules have yet to be fully elucidated, more extensive research can generate new data that can be used in clinical trials. The reported data on these molecules is discussed in detail in the following (Table 2, Figure 1).

#### 3.2.1. *Artemisinin* and Its Derivatives

Artemisinin is a sesquiterpene lactone with a peroxide bridge extracted from the plant *Artemisia annua* [138,139]. Several semi-synthetic derivatives of artemisinin with greater solubility or bioactivity, such as dihydroartemisinin, artemether, and arteether, artesunate have been developed and investigated in several research works [140,141].

Along with its anti-malarial effect, artemisinin and its derivatives have exhibited anti-inflammatory, immunoregulatory, and antioxidant properties [142]. Like some other conventional anti-malarial drugs, including chloroquine and hydroxychloroquine, artemisinin derivatives are assumed to have beneficial therapeutic effects on SLE [143,144]. But in contrast to chloroquine and hydroxychloroquine, which have serious side effects in some cases, no significant side effects have been associated with artemisinin except for mild side effects such as nausea and vomiting or diarrhea [145].

Studies that examined the effectiveness of artemisinin and its derivatives in patients with lupus have shown that long-term use can be effective in improving renal lesions and can prevent recurrence of lupus nephritis. They can relieve the symptoms of patients with SLE. They increased complement levels and also lowered creatinine and urinary protein levels and reduced erythrocyte sedimentation rates [144]. Artesunate increases CD3 and CD4 and increases the CD4/CD8 T lymphocytes ratio. It can regulate the immune function by increasing IL-2 activity and decreasing the level of soluble interleukin-2 receptor (sIL-2R) [144].

Artemisinin can be effective in improving kidney disorders by modulating immune-inflammatory responses. Anti-inflammatory effects of artemisinin are due to its ability to suppress nuclear factor-kB (NF-kB), phosphatidylinositol 3 kinase (PI3K)/protein kinase B (AKT) activity, signal transducer and activator of transcription (STAT), and toll-like receptors (TLRs) [142]. Following the use of artemisinin, the production of proinflammatory cytokines such as TNF-α, IL-6, IL-10, IL-17, and IL-21 is inhibited, but the production of anti-inflammatory cytokines such as IL-4 and IL-10 is increased [146].

Artesunate has suppressed the Jak2-Stat3 signaling pathway in MRL/lpr mice. It has also regulated T follicular helper cell differentiation; thus, it resulted in an increase in follicular regulatory T cells (Tfr) and a decrease in follicular T helper cells (Tfh). It has also reduced the levels of pathogenic cytokines such as IL-6, IFN-γ, and IL-21. It has reduced the level of anti-dsDNA antibodies deposited in the kidney. This means that it might be able to help lessen the symptoms of lupus nephritis [147].

Dihydroartemisinin has been shown to reduce the senescence of myeloid-derived suppressor cells (MDSCs) by regulating the Nrf2/HO-1 pathway. MDSCs are involved in exacerbating the pathogenesis of SLE [148]. Dihydroartemisinin can also restore balance in Treg/Th17 by inducing Foxp3 expression in T cells in mice model [106]. Therefore, dihydroartemisinin is assumed to be effective in improving the condition of SLE patients [148].

##### Toxicity and Side Effects

According to meta-analyses and large clinical studies on artemisinin and its derivatives, they did not demonstrate serious side effects. However, this group of compounds has a number of side effects that could be mentioned, such as neurotoxicity, genotoxicity, hematotoxicity, immunotoxicity, and cardiotoxicity. According to both animal and human studies, artemisinin toxicity is caused by long-term availability rather than by short-term peak concentrations. It is worth mentioning that taking artemisinin orally has a faster rate of elimination than administering it intramuscularly. Therefore, it provides a relatively safe route of administration. This explains why significant toxicities were discovered in the majority of animal research but not in those involving humans [149]. This topic is still open for further research [150].

#### 3.2.2. *Antroquinonol*

Antroquinonol is a derivative of tetrahydro ubiquinone, which was found in the mycelium of *Antrodia camphorata* [151,152]. *A. camphorata* is a mushroom that grows in the inner cavity of the *Cinnamomum kanehirai* (Lauraceae) tree [153] and produces some antroquinonol drivatives, including antroquinonol, antroquinonol B, C, D, L, and M, and 4-acetyantroquinonol B [154]. Hocena is an antroquinonol capsule intended for the treatment of acute myeloid leukemia, hepatocellular carcinoma, and pancreatic cancer and has an orphan drug status from the US Food and Drug Administration [155]. Antroquinonol has been claimed to have the potential to prevent renal disorders and the worsening of lupus nephritis [156]. Inhibiting T cell activation and proliferation, lowering free radical and nitric oxide production, enhancing Nrf2 activation, and decreasing inflammation by inhibiting NF-kB function in the kidney are some of the proposed involved mechanisms [93,156].

In one study, the effect of antroquinonol on preventing the mild form of lupus nephritis from becoming severe was investigated. NZB/NZW F1 mice were used for this purpose and were treated orally with 15 mg/kg antroquinonol for 5 weeks. Eventually, *A. camphorata* reduced hematuria, proteinuria, and IL-18 production in the kidneys. T cell proliferation was also inhibited and Treg cell suppression was induced. Also, reactive oxygen species and nitric oxide production were inhibited, Nrf2 activation was increased, and NF-ĸB activation was inhibited. It was concluded that antroquinonol might be effective in preventing the progression of lupus nephritis [93]. In another study, antroquinonol reduced proteinuria and lowered creatinine and serum BUN levels. It also reduces the thickness of the kidney glomerular basement membrane and inhibits the production of TNF-α and IL-1β. Therefore, the use of *A. camphorata* in autoimmune diseases such as SLE can protect the kidneys [46].

##### Toxicity and Side Effects

In numerous research on animal toxicology, *A. camphorata* exhibited no obvious toxicity. Thus, no significant side effects or deaths were reported, and nausea, vomiting, and diarrhea were the most frequent side effects [157]. Although antroquinonol exhibits cytotoxic activities against cancer cell lines MCF-7, MDA-MB-231, Hep 3B, Hep G2, DU-145, and LNCaP with IC_50_ values ranging from 0.13 to 6.09 μM it is considered safe [158]. Antroquinonol dosages below 30 mg/kg/day do not appear to be associated with any adverse effects [159]. Overall, *A. camphorata* has revealed very little toxicity or side effects in clinical practice.

#### 3.2.3. *Baicalin*

Baicalin is another compound that has a high potential to be considered as a bioactive molecule against SLE. It is a flavonoid isolated from the root of *Scutellaria baicalensis* and has anti-inflammatory and antioxidant effects [99]. Baicalin in MRL/lpr lupus-prone mice has been shown to reduce anti-ds-DNA antibody and urine protein levels. Baicalin has been able to inhibit mTOR activation and also reduce mTOR agonist-mediated Tfh cell expansion and increase Tfr cells. This molecule can inhibit IL-21 production, Tfh cell differentiation, and Foxp3^+^ regulatory T cell differentiation [98]. In a study on pristane-induced lupus in BALB/c mice, baicalin reduced the production of proinflammatory cytokines such as TNF-α, IL-6, IL-10, and IFN-γ. It also inhibited the overproduction of IL-6 and PGE2 and downregulated the aberrant activation of T cells. Thus, it was concluded that baicalin can reduce the severity of SLE and attenuate autoimmunity [99,160].

##### Toxicity and Side Effects

*Scutellaria baicalensis* has long been recognized as a safe and non-toxic herb. *S. baicalensis* oral preparation has no significant side effects; however, some patients may experience stomach discomfort, diarrhea, etc., and those with allergic constitutions may develop a blister-like medication eruption. When used in high doses of injectable preparations, *S. baicalensis* may also result in symptoms such as hypothermia, muscle discomfort, and leucopenia [161]. Some data about possible nephrotoxicity of high doses of baicalin are published, but as a whole, the safety and toxicity of this compound remain still insufficiently studied [162]. Various drug transporters and metabolic enzymes are involved in the disposition of baicalin, and they may be influenced or reciprocally influenced by co-administered medications. These factors can justify the wide herb-drug interactions between baicalin and chemical drugs. Baicalin can significantly alter the pharmacokinetics of medications that have a high protein binding affinity or share the same cytochrome P450 (CYP) enzymes. Phenacetin, theophylline, midazolam, dextromethorphan, nifedipine, and chlorzoxazone can be mentioned among drugs that can interfere with baicalin [163].

#### 3.2.4. Curcumin

Curcumin is the major diarylheptanoid component of turmeric (*Curcuma longa*, Zingiberaceae) [21]. A variety of clinical trials assessing the curcumin effect on inflammation, skin, eye, CNS, respiratory, cardiovascular, gastrointestinal, urogenital, and metabolic disorders have been reported so far [164]. Since curcumin has shown immunomodulatory properties, it has been considered for the improvement of SLE patients. The recommended dosage for SLE ranges from 100–200 mg daily to 4.5 g/day [165]. Curcumin is found to have protective effects against aluminum toxicity and cisplatin-associated neurotoxicity and neuropathy [166,167]. Hypothetically, curcumin may help lupus induced peripheral neuropathy.

The immunomodulatory property of curcumin results from its interaction with various immune mediators, including B and T lymphocytes, macrophage and dendritic cells, cytokines, and various transcription factors such as nuclear factor kappa B (NF-κB), activator protein-1 (AP-1), and signal transducer and activator of transcription (STAT) [168,169,170,171,172,173]. It has been found that curcumin can inhibit the maturation and function of dendritic cells. This function of curcumin is achieved through reducing the expression of MHC-II and co-stimulatory molecules such as CD11c, CD40, CD54, CD80, CD83, CD86, CD252, and CD256. It can also be due to the reduction of proinflammatory cytokines such as IL-1, IL-6, IL-12, IL-12p40, IL-12p70, and TNF-α. In general, curcumin can keep dendritic cells in an immature state, and as a result, it suppresses dendritic cell-mediated stimulation of inflammatory T cells, which play a key role in the severity of symptoms observed in SLE [21].

##### In Vivo and In Vitro Studies

In a study that was conducted on six SLE patients and six healthy individuals, the balance between T helper 17 (Th17) and regulatory T cells (Treg) in SLE patients was investigated. The CD4^+^ cells of these people have been collected, stimulated by Th17 differentiating factors, and exposed to 0.1 and 1 µg/mL of curcumin. Finally, it was found that curcumin can decrease Th17 percentage, decrease IL-17a production, and can increase Treg percentage and increase TGF-β1 production on CD4^+^ T cells of SLE patients. In general, curcumin can modulate the Th17/Treg balance on CD4^+^ T cells of SLE patients without affecting healthy subjects [100].

A study conducted on lupus-prone female MRL/lpr mice has shown that curcumin has the potential to be considered for the treatment of lupus. In this study, mice were treated with 200 mg/kg of curcumin for 8 weeks. As a result, proteinuria, renal inflammation, and spleen size have decreased following the use of curcumin, and a decrease in NLRP3 inflammasome activation was also observed. Following in vitro studies, it has also been found that curcumin can inhibit anti-dsDNA serum induced expression of NLRP3 inflammasome in podocytes [101]. In another study, the ability of oral curcumin consumption to attenuate autoimmunity and renal injury during SLE was evaluated. In order to do this, the female NZBWF1 was given 500 mg/kg/day of curcumin through an oral gavage for 14 days. Finally, it was found that following the consumption of curcumin, weight and body composition were maintained and a decrease in spleen weight and renal injury (glomerulosclerosis) were observed compared to the control group. Ultimately, it has been determined that curcumin can modulate autoimmune activity and probably reduce renal injury in female mice with SLE [102].

In a study, the immune modulation effects of curcumin on pristane-induced lupus mice have been investigated. The female BALB/c mice received an intraperitoneal injection of 0.5 mL pristane for lupus induction. Afterwards, they were treated with 0, 12.5, 50, and 200 mg/kg of bw/day curcumin intragastrically for 16 weeks. As a result, the arthritis score and proteinuria level decreased. However, no significant alteration was observed in body weight. Following 200 mg/kg bw/day curcumin consumption, Th1, Th2, and Th17 percentages decreased, Treg percentages increased slightly, serum IL-6 and IFN-α levels decreased, and antinuclear antibody levels decreased significantly. Therefore, the results have shown that curcumin could be useful as a therapeutic intervention in SLE [103].

##### Toxicity and Side Effects

Long-standing safety data exist for curcumin. For instance, curcumin’s allowable daily intake (ADI) value is 0–3 mg/kg body weight, according to reports from the JECFA and EFSA organizations (the Joint United Nations and World Health Organization Expert Committee on Food Additives and the European Food Safety Authority, respectively) [174]. Despite its well-known safety, several unfavorable side effects have been documented. In a dose-response investigation, seven patients who received 500–12,000 mg and were monitored for 72 h reported symptoms including diarrhea, headache, rash, and yellow stools [175]. In a different study, some participants who received 0.45 to 3.6 g of curcumin per day for one to four months experienced diarrhea, nausea, and a rise in the levels of the enzymes lactate dehydrogenase and alkaline phosphatase in their serum [176].

#### 3.2.5. Emodin

Emodin (1,3,8-trihydroxy-6-methylanthraquinone) is actually a natural anthraquinone that can be found in the barks and roots of many plants, lichens, and molds [177]. One of the main sources of emodin is *Rheum palmatum* (Polygonaceae) which is also known as Chinese rhubarb.

Emodin can reduce steroid resistance by inhibiting P-glycoprotein efflux function. Steroid therapy is part of the common treatment for SLE patients, and a decreased response to steroid therapy following overexpression of p-glycoprotein in peripheral lymphocytes has been observed in some patients [178].

An attempt was made to investigate the effect of emodin on nephritis in a study on BXSB lupus mice. Mice were treated with different doses of emodin for 30 days. As a result, it has been shown that following emodin consumption, the level of proteinuria is reduced and the expression of intercellular adhesion molecule-1 (ICAM 1) in the renal glomerulus is also reduced [107].

The effect of emodin on renal injury in lupus nephritis was investigated. Lupus-prone male BXSB mice were treated with 0, 5, 10, and 20 mg/kg/day emodin for 30 days. Finally, it was observed that following the administration of emodin, glomerular levels of TNF-α, ICAM-1, and fibronectin (FN) decreased, and the levels of urinary protein and serum anti-dsDNA antibody also decreased, and these decreases were dose-dependent. The mechanism of action of emodin is probably through inhibition of dsDNA antibody and decreased levels of TNF-α, ICAM-1, and FN in the glomeruli [108].

##### Toxicity and Side Effects

According to reports, emodin can reduce sperm motility in a dose-dependent manner in mice. Emodin has also been found to have dose- and time-dependent toxicity in kidney and liver cell lines. Intestinal discomfort and severe diarrhea brought on by an overdose of emodin due to its laxative properties lead to an electrolyte imbalance and dehydration [179]. Generally, it is also known to have kidney toxicity, hepatotoxicity, and reproductive toxicity, especially at high doses and long-term use [180] The extremely low bioavailability of emodin further limits its use in therapeutic applications [179].

#### 3.2.6. Esculetin

Esculetin (also known as aesculetin, 6,7-dihydroxycoumarin, and cichorigenin) is a coumarin that has been isolated from a variety of medicinal and toxic plants such as *Cichorium intybus* (chicory) and in *Hydrangea paniculate* Siebold. In a study conducted on MRL/lpr mice, esculetin significantly attenuated renal impairment by reducing BUN, serum creatinine, and albuminuria. Esculetin could improve glomerular hypertrophy and tubular interstitial fibrosis and reduce mononuclear cell infiltration into the interstitium. It was suggested that this molecule could significantly down-regulate the complement cascade as well as the inflammation and fibrosis pathway. In addition, esculetin could up-regulate Nrf2-related antioxidation genes. The authors reported that esculetin could inhibit complement activation both in classical and alternative pathways. The molecule blocked the C3 convertase (C4b2a) to exert this inhibitory capability. Moreover, it was suggested that the antioxidation effect of esculetin was dependent on Nrf2 activation, which means that esculetin could inhibit NFκB nuclear translocation and TGFβ-smad3 profibrosis pathway [109]. Lupus nephritis is one of the important complications of lupus, and complement activation contributes to kidney injury; the inhibition of complement activation by herbal compounds might be beneficial for lupus. It was also reported that the coumarin derivates that are isolated from *H. paniculata* could improve renal injuries in cationized-BSA-induced membranous nephropathy. The suggested mechanism was the inhibition of complement activation and interleukin 10-mediated interstitial fibrosis [181].

##### Toxicity and Side Effects of Esculetin

Acute toxicity studies reported LD_50_ for intraperitoneal injection to mice as 1450 mg/kg and >2000 mg/kg by mouth. No reported adverse effects are known other than LD_50_ [182].

#### 3.2.7. Mangiferin

The main source of mangiferin is *Mangifera indica*, although it is found in 96 species, 28 genera, and 19 families of angiospermic plants. *Mangifera indica* belongs to the family Anacardiaceae and is known as mango. Almost all parts of *M. indica*, such as fruits, twigs, leaves, and stem bark, contain mangiferin [183]. Mangiferin is a xanthonoid polyphenol with a variety of pharmacological effects such as anti-inflammatory, antioxidant, immunomodulatory, nephroprotective, hepatoprotective, anti-cancer, anti-diabetic, and anti-asthma [184]. According to certain research, its renal protective actions may be beneficial for those with lupus nephritis [166,167].

Mangiferin has been shown to improve lupus nephritis in lupus-prone B6/gld mice. In a study, the effect of mangiferin on lupus nephritis was investigated. Mice were treated orally with 20 or 40 mg/kg/day of mangiferin for 12 weeks. Finally, Mangiferin has been shown to be effective in treating lupus nephritis with its anti-inflammatory and immunomodulatory effects. Mangiferin was effective by suppressing mTOR signaling pathways, upregulating CD4^+^ FoxP3^+^ Tregs, and inhibiting T cell proliferation. Mangiferin improved renal immunopathology and reduced renal T cell infiltration. It also lowered serum creatinine and urinary protein levels and increased CD4^+^ FoxP3^+^ Treg frequencies in the spleens, lymph nodes, and kidneys [113].

##### Toxicity and Side Effects

Mangiferin is typically regarded as a non-toxic natural substance. Adults receiving 0.9 g of mangiferin orally demonstrated no toxicity. LD_50_ of the mangiferin was considered to be 400 mg/kg on mice [185]. Mangiferin was found to be safe and helpful in enhancing cellular function, according to numerous research works [186]. In a study that assessed the toxicity of mango leaf extract, which was given orally to rats for three months at a dose of 2 g/kg body weight per day, neither mortality nor toxic effects were observed [187]. The *Mangifera indica* leaf aqueous extract was not particularly mutagenic or genotoxic. Mangiferin has generally been shown to be safe in cell and animal research. In contrast, there are insufficient safety data from human research [186].

#### 3.2.8. Salvianolic Acid A

Salvianolic acid A (or Dan phenolic acid A) is a phenolic compound extracted from *Salvia miltiorrhiza* (Lamiaceae family). The plant is also known as Chinese sage, Danshen, and red sage. Salvia species such as *S. officinalis* and *S. miltiorrhiza* have shown antioxidant, antibacterial, anti-cancer, and anti-diarrheal effects and have been used to treat lupus and autism, lower cholesterol, treat Alzheimer’s, reduce sweating, and reduce menopausal hot flashes [188].

In a study performed on BALB/c mice, the effect of salvianolic acid A isolated from the root of *S. miltiorrhiza*, on lupus nephritis was investigated. Mice were treated with 5 mg/kg/day of salvianolic acid A for 5 months. As a result, it was observed that following the consumption of salvianolic acid A, anti-Sm autoantibodies decreased, phosphorylation of IKK, IκB, and NFκB in kidney tissue was inhibited, and pathological effects were reduced [114].

##### Toxicity and Side Effects

In an acute toxicity study, the LD_50_ of salvianolic acid A was reported as 1161.2 mg/kg in mice. In dogs’ animal model, the minimum lethal dose and maximal non-lethal dose of salvianolic acid A were reported as 682 mg/kg and 455 mg/kg in dogs, respectively. Based on a 4-week repeated-dose, no observed adverse effect level was 20 mg/kg. It was suggested to examine liver and kidney function during the administration of salvianolic acid A in a clinic [189]. According to a system review of the drug’s safety, the clinical use of salvianolate injection did not result in the occurrence of any common or major side effects. Blood loss and allergic reactions are the most common adverse effects of salvianolic acid injections. In general, it has been determined that salvianolic acid is well tolerated in the general population. Rash, erythemas, pruritus, palpitations, headaches, dizziness, elevated blood bilirubin, elevated transaminases, elevated blood creatinine, positive fecal occult blood, and abnormal platelet count are among the most common adverse effects that were reported for salvianolic acids [190].

#### 3.2.9. Triptolide

Triptolide is a diterpene triepoxide isolated from *Tripterygium wilfordii* Hook F (*T. wilfordii*). The molecule has immunosuppressive and anti-inflammatory effects and has been shown to have therapeutic effects on autoimmune and inflammatory diseases such as lupus nephritis, arthritis, neurodegenerative disorders, and asthma [23,139]. Despite the beneficial effects of Triptolide in the treatment of various inflammatory disorders, it should be noted that the use of *T. wilfordii* can cause severe toxicity and side effects. This may limit the clinical use of this plant [191]. Triptolide’s anti-inflammatory and immunosuppressive effects are due to its ability to inhibit the proliferation of immune cells and inflammation-related cells and reduce cytokines and proinflammatory mediators [192].

The effects of triptolide on SLE are assumed to be through induction of miR-125a-5p and an increase in the proportion of Treg [124]. Triptolide has been shown to reduce the expression of transforming growth factor-beta (TGF-β) and vascular cell adhesion molecule (VCAM-1) [193]. It can also reduce the expression of C3 and CD40, so it generally has immunosuppressive and anti-inflammatory effects and is useful in renal disorders [194]. Another way triptolide can be immunosuppressive and anti-inflammatory is through changing signaling pathways. Triptolide has been reported to inhibit nuclear factor-κB (NF-κB) signaling pathway [195], lower the IL-17 level, and suppress IL-6/signal transducer and transcription 3 (STAT3) signaling pathway [196].

(5R)-5-Hydroxytriptolide (LLDT-8) is a triptolide analogue. It has strong anti-inflammatory and immunosuppressive activity [196]. LLDT-8 improves anti-GBM glomerulonephritis because it can regulate Fcγ signaling pathway [197]. It can also improve lupus nephritis and reduce the infiltration of kidney immune cells because it inhibits the expression of renal chemokines [125].

##### Toxicity and Side Effects

The subject of triptolide‘s safety in clinical applications has been brought up because of its broad usage. Despite the valuable pharmacological effects of triptolide, its application requires particular caution because it is well known to have hepatotoxicity, nephrotoxicity, reproductive toxicity, etc. [198]. Hepatic cytochrome P450s are involved in the metabolism of triptolide, and triptolide toxicity and CPY3A also have a close relationship. Clinical case reports have shown through research that triptolide exposure can be involved in damaging a variety of organs, including the kidney, liver, heart, ovary, and testicles. Additionally, it has been shown that triptolide has a variety of harmful effects on cells, including damage to membranes, oxidative stress, endoplasmic reticulum stress, metabolism dysfunction, mitochondrial dysfunction, apoptosis, and autophagy [199].

#### 3.2.10. Total Glycosides of Paeony (TGP)

Total glycosides of paeony (TGP) are extracted from the root of *Paeonia lactiflora*. TGP has long been used to treat autoimmune diseases [200]. The beneficial effects of TGP on lupus patients are dependent on its anti-inflammatory and immunosuppressive effects [201]. The effects of TGP on the production of proinflammatory cytokines, antibody production, apoptosis of lymphocytes, and lymphocyte proliferation are dual and dose-dependent [202]. TGP increased the mouse splenocytes’ proliferation at low doses (0.05~0.4 mg/L), while it decreased it at high doses (0.4~1.6 mg/L) [202,203,204]. The ratio of T helper cells to T suppressor cells (Th/Ts) increases at low doses of TGP (0.2 mg/L) and decreases at high doses of TGP (6.0 mg/L) [204]. IL-1 production increases at low doses of TGP (0.5~12.5 mg/L) and decreases at high doses of TGP (12.5~312.5 mg/L) [203]. IgM-antibody production increases at low doses of TGP (0.1~0.4 mg/L) and decreases at high doses of TGP (0.4~3.2 mg/L) [205,206]. Therefore, it is assumed that the immunomodulatory effects of TGP are dose-dependent, and the dose should be adjusted for best results.

The beneficial effect of TGP on SLE has been discussed in several studies. It was reported that the anti-inflammatory effect of TGP is due to its ability to inhibit the production of nitric oxide, leukotriene B4, and prostaglandin E2 [202]. TGP reduces the SLEDAI score in SLE patients and also reduces the average daily dose of prednisolone [115]. A decrease in renal pathology has been observed following the consumption of TGP by MRL/lpr mice. TGP has also reduced the levels of anti-dsDNA antibodies and antinuclear antibodies (ANA). It could also reduce urinary protein levels. Consequently, it was concluded that TGP consumption in patients with lupus nephritis can have therapeutic effects [119]. TGP causes down-regulated Foxp3 promoter methylation levels, thus increasing the expression of Foxp3 in lupus CD4^+^ T cells. TGP increased the number and percentage of Treg cells in lupus CD4^+^ T cells and increased IFN-γ and IL-2 expression [200]. TGP increases DNA methylation of ITGAL promoter in CD4^+^ T cells, thereby reducing CD11a gene expression [120].

##### Toxicity and Side Effects

In general, TGP is considered a safe and effective compound that is tolerable and does not cause any serious side effects. According to studies, the likelihood of developing diarrhea after consuming TGP may rise. TGP can accelerate the gastrointestinal tract’s peristalsis, which may be the cause of the diarrhea. The majority of patients only experience moderate and acceptable symptoms, and the gastrointestinal system is not organically harmed. Drowsiness, dry mouth, dizziness, and weakness are some additional side effects that have been reported [207].

### 3.3. Fatty Acids, Vitamins, and Minerals

Certain nutrients and vitamins as dietary supplements have been consumed to improve lupus [208]. The efficacy of some of them have been investigated and discussed in several studies (Table 2). Safety and side effects of fatty acids, vitamins, and minerals is well studied by numerous publications and are available even on the indications of these over-the-counter (OTC) medicines.

#### 3.3.1. Fatty Acids

Unsaturated oils play an important role in the immune system regulation. In human nutrition and/or healthcare, seed oils have long been utilized as a daily supplement, a food ingredient, or a therapeutic cure. Long chain fatty acids (LCFAs) are fatty acids with more than 14 carbons and make up the majority of vegetable oils. They are necessary for the human body’s ongoing regular cell growth and development. Among these, polyunsaturated fatty acids (PUFA) like n-3 and n-6 fatty acids are crucial for the prevention and treatment of many chronic diseases, including diabetes, coronary artery disease, inflammatory and autoimmune disorders, and many other ailments. Some significant fatty acids, such as linoleic acid (an n-6 fatty acid) found in the majority of vegetable oils and plant seeds, are regarded as essential fatty acids (EFAs). Arachidonic acid, which can be further elongated and desaturated to form prostaglandins, thromboxanes, and leukotrienes, is one of these EFAs. A different class of EFA is the n-3 fatty acids, which include linolenic acid and are present in soy, linseed, and flaxseed oils. According to some evidence, n-3 fatty acids have protective effects on eicosanoid metabolism. Docosahexaenoic acid (DHA), a crucial component of cellular membranes and another significant n-3 fatty acid, has a favorable impact on coronary heart disease, inflammatory disease, atherosclerosis, and disorders of the nervous system [209]. Dietary lipids are also involved in autoimmune phenomena by affecting the balance between Th1 and Th2 cells [210,211].

Dysregulation of PUFAs induces a wide range of neurological and developmental disorders. Linoleic acid and linolenic acid are required as part of the immune cell membrane [212]. α-linolenic acid and γ-linolenic acid are among the omega-3 acids that have beneficial effects following the reduction of TNF-α and IL-2 in SLE patients. Omega-3 fatty acid supplementation has shown potential benefit on SLE disease activity as demonstrated by Systemic Lupus Activity Measure-Revised (SLAM-R), SLE Disease Activity Index (SLEDAI), and British Isles Lupus Assessment Group (BILAG) scores as well as plasma membrane arachidonic acid composition and urinary 8-isoprostane levels, with minimal adverse effects [213].

Finding the optimal ratio of ω-6/ω-3 PUFAs is essential in therapeutic interventions. As an example, linoleic/alpha-linolenic of 1:3 is the optimal ratio for enhancing both the proliferation and differentiation of cells such as neural stem cells [214]. Wei et al. concluded in a meta-analysis that low-ratio n-6/n-3 PUFA supplementation could significantly reduce serum TNF-α and IL-6 concentrations but not CRP concentrations [215].

In the NZB × NZW mice animal models, essential fatty acid deficient diets can reduce arachidonic acid levels, thus reducing proinflammatory prostaglandins and leukotrienes, and also reduce nephritis by inhibiting autoantibody production [212]. Studies have shown that the lifespan increased and autoantibody levels decreased in animal models of SLE following a diet rich in omega-3 fatty acids [22].

The presence of omega-3 PUFA in the diet of SLE patients can regulate blood pressure and proteinuria and also reduce anti-dsDNA levels, as well as TNF-α, IL-1α, IL-1β, and IL-2 [126,213].

A meta-analysis conducted in 2020 found that omega-3 fatty acids could reduce SLE activity. In this study, 136 patients in the comparison group and 138 in the treatment group were used, and the mean age of patients was 43 years. The follow-up time of the trial varied between 12 and 52 weeks. This study showed that the use of omega-3 fatty acids is more effective than placebo in reducing disease activity in SLE [22].

Eicosapentaenoic (EPA) and docosahexaenoic (DHA) are some of the unsaturated fatty acids that exert their anti-inflammatory effects by lowering the level of C reactive protein (CRP) and other inflammatory mediators [126,216,217]. The most widely available dietary source of EPA and DHA is cold-water oily fish, such as salmon, herring, mackerel, anchovies, and sardines.

EPA and DHA can affect the immune system through various mechanisms. They can inhibit the enzyme lipoxygenase and subsequently reduce the inflammatory factors derived from arachidonic acid. DHA can inhibit nuclear factor κB (NF-κB) and TNF-α [218].

DHA has increased the lifespan of and suppressed glomerulonephritis in NZB × NZW mice with systemic lupus erythematosus, possibly due to inhibition of IL-18 induction [126]. DHA has also reduced IL-18 levels, lowered serum levels of anti-dsDNA, and regulated IgG renal deposition in mice [126,219].

#### 3.3.2. Vitamin A

Some studies have been conducted to investigate the effectiveness of vitamin A in lupus. Retinoic acid is a metabolite of vitamin A. Vitamin A deficiency in lupus patients has been shown to have a negative effect on the prognosis of the disease. Consumption of retinoic acid and vitamin A regulates the balance between Th17 and Treg. It was reported that following the intake of vitamin A by lupus patients, the level of Th17 decreased and the level of Treg increased [127,128].

#### 3.3.3. Vitamin B

Vitamins B6, B12, and folate reduce homocysteine levels, so they can be helpful in improving atherosclerosis in SLE patients. They can also lower levels of inflammatory cytokines and C-reactive protein (CRP). Vitamin B6 can also reduce the risk of active disease by lowering homocysteine [129]. Following the use of niacin, a decrease in triglyceride and LDL-C levels was observed, with no significant effect on HDL-C levels [130]. In general, it was suggested that taking supplements of the vitamin B complex could be beneficial for people with SLE.

#### 3.3.4. Vitamin C

Vitamin C has an antioxidant effect. It can release inflammatory mediators and modulate immune function. It also lowers anti-dsDNA levels and IgG. Vitamin C can prevent active SLE [131]. Concomitant use of 500 mg of vitamin C and 800 mg of vitamin E daily for 3 months has shown a slight decrease in lipid peroxidation. In SLE patients with high doses of vitamin C, ascorbate is found in the urine, so the maximum dose of vitamin C is 1000 mg/day [220].

#### 3.3.5. Vitamin D

It has been shown that there is a link between vitamin D deficiency and the severity of SLE. Higher SLEDAI scores have been reported in patients with low levels of vitamin D. Supplementation with vitamin D in SLE patients inhibits dendritic cell activation and maturation [132]. Calcitriol is the active form of vitamin D and acts on autoimmune diseases such as SLE by regulating the response of T and B cells and boosting the innate immune response [133]. SLE patients are photosensitive and should use sunscreen when exposed to the sun. On the other hand, sunlight is needed to produce vitamin D, so it can be assumed that taking vitamin D supplements might be useful for SLE patients [221,222].

#### 3.3.6. Vitamin E

Vitamin E has antioxidant and anti-inflammatory effects and, because of its anti-inflammatory effect, seeks to reduce IL-2, IL-4, and TNF-α, which can be effective in lupus [223]. Furthermore, vitamin E consumption by SLE patients reduces the generation of autoantibodies [134].

#### 3.3.7. Calcium

In some SLE patients, a decrease in bone mineral density has been observed, which may or may not be dependent on corticosteroid use. On the other hand, these patients are mostly deficient in vitamin D and avoid exposure to sunlight. Therefore, adequate calcium intake is important for SLE patients [223].

#### 3.3.8. Iron

There should be a balance of iron intake in SLE patients. Iron supplementation to MRL/MPJ-lpr/lpr mice resulted in cell damage, renal lesions, and worsened renal impairment in an in vivo study. Iron chelators have also been shown to be beneficial in autoimmune diseases. In contrast, iron deficiency increases the symptoms of the disease, so iron should be used in SLE patients who have anemia [135,224].

#### 3.3.9. Selenium

Selenium supplementation has been considered in the treatment of lupus because of its antioxidant and anti-inflammatory effects. A study on NZB/NZW female mice found that survival increased following selenium supplementation, which may be due to increased levels of natural killer cell activity [136]. In an in vitro study performed on the B6.Sle1b mouse model of lupus, an attempt was made to find the mechanism of the effect of selenium on lupus. It has been shown that selenium can inhibit the activation, differentiation, and maturation of macrophages and B cells. Therefore, its use can be useful in patients with lupus [137]. Reduced selenium levels have been observed in patients with autoimmune diseases, which may be considered a risk factor for the onset of autoimmunity and inflammation. Due to the anti-inflammatory effect of selenium, it has been suggested that consuming a certain amount of selenium in patients with autoimmune diseases can lead to better management of disease complications [225].

#### 3.3.10. Zinc

It was shown that a zinc-restricted diet can increase serum levels of corticosteroids and subsequently reduce the symptoms of autoimmune diseases such as SLE, so it can be useful in controlling SLE [226]. A study of NZB/NZW mice showed a decrease in autoantibody production. In MRL/lpr mice, the use of zinc-restricted diets decreased the level of anti-dsDNA, decreased lymphoproliferation, and recovered glomerulonephritis [210]. On the other hand, it should be noted that a study conducted on humans has shown that zinc deficiency causes immune dysfunction by acting on Th cells and can lead to neurosensory disorders and reduced body mass [227].

### 3.4. Herbal Medicines, Medicinal Plants, Mushrooms, and Fungi and Their Crude Extracts

A variety of medicinal plants and mushrooms have been reported to exhibit efficacy against lupus conditions (Table 3). In some traditional remedies, they have been used in the form of dried powdered plant material or fungi. In some others, a crude extract of the plant or fungus was prepared using solvents such as water or ethanol or a mixture of both (hydroalcoholic extract). Crude extracts are a complex mixture of molecules with the same range of polarity but different concentrations. Sometimes, several molecules with a variety of mechanisms work synergistically to produce a specific effect. Although the crude extracts of herbal medicine can reflect the efficacy of a special herb or fungi, due to the variation of compounds in the natural sources, special attention should be given to the standardization and authentication of them in order to have repeatable and reliable effects. Compared to purified bioactive molecules, crude extracts usually exhibit milder efficacy and side effects, and introducing supplements from herbal medicine to the market is much easier.

The use of herbal medicines has long been used to treat various diseases, such as SLE. In this article, an attempt has been made to review effective herbs for improving lupus. The use of traditional medicine along with Western medicine can reduce the dose of Western medicine drugs, reduce their side effects, and ultimately improve the quality of life of SLE patients.

#### 3.4.1. *Tripterygium wilfordii* Hook. F.

*Tripterygium wilfordii* Hook. F. (Celastraceae) has the common names of “thunder duke vine” and “thunder god vine” and is also known as “léi gōng téng” in Mandarin. The plant has long been used in traditional Chinese medicine (TCM) and it is widespread in southern and eastern China [88,253]. Its root extract is used, but its bark must be removed because of its toxicity. There are various compounds with a range of biological effects in the root extract, and the procedures and methods of extraction play a role in overcoming the plants’ toxicity [254,255]. In TCM, the *T. wilfordii* preparations have long been used for some health conditions, including sores and swelling, inflammations, ankylosing spondylitis, hepatitis, nephropathy, allergic skin diseases, inflammatory lesions of leprosy, and cancer [253,254,256,257,258,259]. The effect of *T. wilfordii* on autoimmune diseases such as SLE, rheumatoid arthritis (RA), BehCet’s disease, psoriasis, etc., has been investigated in several studies [253,260]. Its effect on kidney transplantation, nephrotic syndrome, and diabetic nephropathy has also been investigated [261,262,263].

##### Clinical Trials

A number of studies have examined the efficacy of *T. wilfordii* in lupus erythematosus. A clinical trial study compared the effect of *T. wilfordii* with prednisolone. In this study, there were 23 cases of lupus erythematosus, of which 15 had SLE and 8 had discoid lupus erythematosus (DLE). They had been given 45 mg/day of crude extract of *T. wilfordii*. The control group consisted of 19 cases of SLE treated with prednisolone. The rate of improvement was almost the same in the two groups, and no significant difference was observed. However, there are benefits to using *T. wilfordii*, including the recovery from erythematosus rash and arthralgia [249]. The immunomodulatory and anti-inflammatory effects of *T. wilfordii* have also been reported in a clinical study on 26 DLE cases [250].

Despite the benefits of this plant, side effects have also been reported. Headaches, gastrointestinal complications, nausea, diarrhea, infertility, etc., are among these adverse effects [249,250] One of the side effects reported due to long-term use of *T. wilfordii* in women is decreased bone mineral density [121]. Due to some toxic side effects of *T. wilfordii* such as kidney damage, its use is not recommended in SLE patients who also have nephropathy [249]. A systematic review has discussed cardiovascular, hematologic, and skin complications as well as infertility and gastrointestinal complications of this plant [122]). Side effects are more common with high concentrations. Consuming the right amounts and rational treatment will help control the side effects and obtain therapeutic effects [121].

##### Active Compounds and Possible Mechanisms of Efficacy

A variety of compounds have been isolated from *T. wilfordii* such as triptonide, tripodine, triptolide (TPT), tripdiolide (TPO), etc. [123]. Among the phytochemicals of *T. wilfordii* root extract, celastrol (tripterine) and triptolide (diterpenoid triepoxide) are the most investigated [124,258]. Triptolide’s anti-inflammatory and immunosuppressive effects are mediated by inhibition of T cells and inhibition of IL-17 and STAT3 transcription [125]. It has been observed that NF-κB activity is significantly reduced in patients with SLE after consuming *T. wilfordii.* It was suggested that *T. wilfordii* may exert an immunosuppressive effect on SLE patients by inhibiting NF-κB expression [123].

An in vitro study on dendritic cells (DCs) of SLE patients has shown that triptolide can inhibit the differentiation and maturation of DCs and reduce the immune function of DCs. In this study, doses of 0, 5, 10, and 30 μg/L of triptolide were used. Triptolide also reduced secretion of IFN-α, IL-6, and TNF-α [121]. In another study, to evaluate the effect of triptolide, BALB/c-un nude mice were used. Mice were treated orally with 5 mg/kg/d of triptolide, and their blood samples were collected before treatment and 1, 3, and 6 months after treatment. Finally, it was found that with the use of triptolide, a decrease in the percentage of CD8^+^, Tcl, Thl cells, CD4^+^/CD8^+^, Thl/Th2, and Tcl/Tc2 and an increase in the percentage of CD4^+^, Tc2, and Th2 cells was observed [122].

The effect of triptolide and tripdiolide on lupus nephritis in (NZB×NZW) F1 mice has been investigated. Mice were treated orally with 6 µg of triptolide or tripdiolide for 15 weeks. Finally, triptolide and tripdiolide have been shown to reduce BUN, proteinuria, and anti-dsDNA antibody levels, as well as the production of cytokines such as IL-6 and TNF and monocyte chemoattractant protein 1. Therefore, triptolide and tripdiolide have therapeutic effects in lupus nephritis [123].

In a study, the effect of triptolide on SLE was investigated in female MRL/lpr mice treated with 0.2 or 0.3 mg/kg/d of triptolide for 13 weeks. Compared to the control (vehicle) group, triptolide significantly reduced proteinuria, serum anti-dsDNA, and renal histopathologic assessment. The effect was comparable to that of cyclophosphamide (20 mg/kg/w). Triptolide also increased the proportion of Treg and induced expression of miR-125a-5p [124].

(5R)-5-Hydroxytriptolide (LLDT-8) is a triptolide derivative that has strong anti-inflammatory and immunosuppressive effects and low toxicity. In a study, the effect of LLDT-8 on lupus nephritis was investigated. Female MRL/lpr mice were treated with 0.125 mg/kg/2 days of LLDT-8 for 9 weeks. Finally, LLDT-8 has been shown to reduce proteinuria, serum creatinine, and glomerular IgG deposits, and it could also ameliorate histopathology and increase the lifespan of mice. LLDT-8 reduced the expression of inflammatory cytokines such as IFN-γ, IL-17, IL-6, and TNF-α and inhibited immune cell infiltration in the kidneys. It was suggested that LLDT-8 could have therapeutic effects on lupus nephritis [154].

##### Toxicity and Side Effects

Refer to the section on toxicity and side effects of triptolide in this text.

#### 3.4.2. *Ophiocordyceps sinensis* (syn. *Cordyceps sinensis*)

*Ophiocordyceps sinensis* belongs to the Ophiocordycipitaceae family, is an entomogenous fungus used in TCM. It is also known as Yartsa gumba or caterpillar fungus [79,264].

##### Clinical Trials

Several clinical trials have evaluated the efficacy of the dry powder of *O. sinensis* mycelium (Bailing capsules) as a supplement in conjunction with prednisolone, cyclophosphamide, tacrolimus, or leflunomide on lupus nephritis. They have reported controversial results, which might be due to the small sample size or differences in control groups and study design [142]. A meta-analysis study was conducted on a total of 14 studies comprising 1301 participants, which were combined for analysis in the present study. In general, this study showed that consumption of *O. sinensis* mycelium (Bailing capsule) for lupus nephritis is more effective than not using it. Although there was no significant difference between the Bailing group and the control group in anti-ds-DNAIgM levels and complement C3 levels (which is associated with the existence of some immune diseases like SLE), other indicators of the disease, such as SLEDAI score, Alb, 24 h urinary protein, serum creatinine, and the number of effective treatments and complications, improved. It was concluded that *O. sinensis* could be beneficial in the treatment of lupus nephritis [142].

In a study of 61 lupus nephritis patients, the prevention of the recurrence of lupus nephritis by *artemisinin* and *C. sinensis* was evaluated. A total of 30 patients were in the control group, and 31 patients were treated with 2–4 g/d of *C. sinensis* and 0.6 g/d of artemisinin. *C. sinensis* was taken before three main meals and artemisinin after three main meals for three years. The control group took tripterygiitotorum and/or Baoshenkang. Finally, the creatinine clearance rate did not change between before and after treatment, and the complement C3 level stabilized in the normal range. It was concluded that the combination of *C. sinensis* and artemisinin could be effective in the prevention of recurrence of lupus nephritis [95].

##### Active Compounds and Possible Mechanisms of Efficacy

A variety of bioactive phytochemicals with biological activities have been reported from *O. sinensis*, such as cordycepin, cordycepic acid, polysaccharides, ergosterol, nucleosides, fatty acids, proteins, minerals, etc. The immunomodulatory effects of *O. sinensis* were mostly attributed to its polysaccharides, which affect both humoral and cellular immune responses in vivo and improve the serum levels of ovalbumin-specific IgG, IgG1, and IgG2b levels. This fungus’s mycelia polysaccharides have been shown to enhance the proliferation and phagocytosis of macrophages and stimulate macrophages [265]. Intracellular polysaccharides isolated from submerged cultures of *O. sinensis* have been reported to exhibit strong immunomodulatory effects on RAW264.7 macrophage cells via the MAPK and PI3K/Akt signaling pathways. It enhanced the phagocytic activity of RAW264.7 cells and increased cytokine production. This immunomodulatory response was mediated by the secretion of both proinflammatory cytokines (TNF-α, IL-6, and IL-1β) and anti-inflammatory substances (TGF-β1 and IL-10), producing NO and promoting the expression of iNOS [266].

Some of *O. sinensis*’ nucleotide contents have also been reported to exhibit immunomodulatory properties by lowering NO and increasing IL-lβ and TNF-α release from macrophages [265]. Deoxynucleic acids from *O. sinensis* activated mouse bone marrow-derived DCs via a toll-like receptor 9-dependent pathway [265]. Aside from the total extracts of *O. sinensis*, specific components, including 1-(5-Hydroxymethyl-2-furyl)-β-carboline, and cordymin (a purified peptide from *O. sinensis*), displayed significant anti-inflammatory properties [79,267,268,269].

Hypothetically, besides immunomodulatory and anti-inflammatory effects, the antioxidant [270], cardiovascular [271], and kidney protective [272] properties of *O. sinensis* may also have a role in the beneficial effects of this fungus on SLE.

A number of studies have been performed to investigate the effect of *O. sinensis* on lupus. A study was performed on lupus-prone (NZB/NZW) F1 hybrid mice. The mice were divided into four groups of different ages (three, six, and eight months) and were given 2.4 mg/g/day of cultured mycelia of *C. sinensis* orally. The fourth group was also used as a control. The results showed that in groups who started taking it at the ages of 3 and 6 months, survival increased, proteinuria decreased, and titers of anti-double-stranded DNA antibodies decreased. The percentage of CD4^+^ T cells in peripheral blood mononuclear cells (PBMC) decreased significantly, while the percentage of CD8^+^ T cells increased. Eventually, the results of this study showed that early administration of C. sinensis reduces the severity of lupus disease [242]. In another study on MRL lpr/lpr mice, a triterpenoid, component H1-A, was extracted from *C. sinensis*. Administration of 40 µg/kg/d of H1-A daily for 8 weeks to mice aged 12 weeks resulted in a reduction in the production of anti-dsDNA, lymphadenopathy, and proteinuria. Renal function has improved, and no significant changes have been observed in immune complex deposition. In general, H1-A intake has increased the survival of mice with lupus [96].

In another study, the effects of Chinese herbs on SLE were investigated in NZB/NZW F1 mice at one month of age. *C. sinensis* was found to inhibit anti-ds-DNA production and increase the lifespan of mice. Although *A. sinensis* does not inhibit anti-ds-DNA production, it has been able to increase the lifespan of mice [243].

##### Toxicity and Side Effects

In addition to the claimed therapeutic or positive effects for the chemicals derived from the Cordyceps fungi, cytotoxicity and/or neurological toxicity adverse effects have also been described for these compounds. After daily intake of Cordyceps fruiting bodies or associated products, reports of nausea, diarrhea, and even significant post-extraction bleeding have been documented. There have also been a few rare reports of dry mouth, nausea, and diarrhea [273].

#### 3.4.3. *Ganoderma Lucidum* and *Ganoderma Tsugae*

*Ganoderma lucidum* belongs to the family Ganodermataceae or Polyporaceae and is also known as Lingzhi or Reishi. This fungus has been observed to have anti-inflammatory, antioxidant, and analgesic effects, and it has been used in TMC since the ancient era. This fungus is used for various diseases that, in most cases, have an inflammatory basis, such as arthritis, hepatitis, bronchitis, acute colitis, etc., as well as hypertension and malignancy [236].

*G. lucidum* has shown an immunomodulatory effect on PMNC (peripheral mononuclear cells). It has exhibited suppressive effects on tumor necrosis factor-α (TNF-α), IL-1β, IL-12, and IL-6, which are pathogenic cytokines associated with SLE [274,275].

##### Clinical Trials

The authors of the present study could not find a clinical trial on the efficacy of the *G. lucidum* on SLE, but there are several trials investigating the efficacy of this mushroom on some other conditions such as rheumatoid arthritis [276], fibromyalgia [111,277], neurasthenia [278], cancers [279], cardiovascular risk factors of metabolic syndrome [280], lower urinary tract symptoms (LUTS) [281], etc. Moreover, in a clinical trial, β-glucans of *G. lucidum* were reported as safe and well-tolerated immunomodulator supplements for children [282].

##### Active Compounds and Possible Mechanisms of Efficacy

A variety of natural polysaccharides have shown immunomodulatory, anti-inflammatory, and wound-healing properties [283,284]. *G. lucidum* polysaccharides (GLPs), such as ganoderan and β-glucans, have been extensively studied for their biological activities, which include antioxidant, antitumor, anti-inflammatory, anti-diabetes, and immunomodulatory properties [285]. GLPs can affect different immune effector cells, including lymphocytes and myeloid cells. They can modulate innate immunity, cellular immunity, and humoral immunity [286]. Also, some triterpenoids of *G. lucidum* (GLTs), such as ganosidone A and its derivatives [287], as well as ganoderic acid D [288], and 3-oxo-5α -lanosta-8, 24-dien-21-oic acid [289] have shown anti-inflammatory effects.

In a study, the combination of *G. lucidum* and *San-Miao-San* (SMS) has been used to evaluate the anti-inflammatory effect on SLE. SMS is a Chinese herbal medicine that consists of a combination of three herbs that include Phellodendri Cortex (Huangbai), Atractylodes Rhizome (Cangzhu), and Radix achyranthis bidentatae (Niuxi). The control group consisted of female Balb/c mice (at the age of 20–24 weeks). The study group included three groups of female MRL/lpr mice that had mild, moderate, and severe lupus. Initially, 500 mg/kg/day was administered orally for 7 days, and then 50 mg/kg/day was injected intraperitoneally for 7 days. Finally, a significant reduction in anti-ds-DNA in the study group with moderate and severe SLEs was observed. In the study group, the percentages of IL-10, CD4^+^, CD25^+^, Foxp3^+^ and Treg cells increased significantly, the concentrations of IL-2 and IL-12P70 increased significantly, and the concentrations of IL-21, IL-10, and IL-17A decreased significantly [236].

In a study, the effectiveness of *Ganoderma tsugae* on increasing the lifespan of NZB/NZW F1 mice was investigated. All groups of mice (two months of age) were given standard laboratory chow feeding. The first study group was given 0.1 cm^3^ of oral ganoderma extract, and the second study group was given 0.2 cm^3^ of oral ganoderma extract daily. The third study group was given 0.5 mg/kg/day of prednisolone. *G. tsugae* increased the life expectancy in mice with lupus and reduced anti-dsDNA autoantibody and proteinuria, as well as parenchyma and perivascular mononuclear cell infiltration [237].

##### Toxicity and Side Effects

Patients from various nations have reported developing human sensitivity to the *G. lucidum* antigen. Since *G. lucidum* has an anticoagulant effect and extending the prothrombin time has an additional effect on clotting factors, patients who were taking anticoagulants or antiplatelets should take caution. Hypoglycemic people should also take caution because it reduces blood sugar levels. *G. lucidum* is an anti-hypertensive agent, according to numerous research works. Before using it, persons with cardiac issues should visit a physician [290]. In a study, it has been determined that sub-chronic toxicity of the liver occurs when rats are given more than 1.2 mg/kg body weight of *G. lucidum* extract [291].

#### 3.4.4. *Urtica Dioica* L.

*Urtica dioica*, also known as stinging nettle, grande ortie, or anonhasquara, belongs to the Urticaceae family. Different parts of the plant, such as roots, leaves, seeds, and aerial parts, have different therapeutic effects upon extraction by different methods. Following animal studies, various therapeutic effects without the appearance of serious side effects have been reported. This plant has shown anti-inflammatory, antioxidant, antimicrobial, antifungal, etc., effects. In traditional medicine and ethnomedicine, different parts of the plant have been used in diseases such as systemic lupus erythematosus and rheumatoid arthritis, diabetes, prostate cancer, breast cancer, atherosclerosis, cardiovascular diseases, etc. Non-aqueous extraction of the root of this plant has been found to be effective in SLE [292,293].

##### Clinical Trials

In a case report, one female lupus patient (24 years old) with a renal allograft status and elevated serum creatinine was featured. The patient was consuming immunosuppressants (prednisone, CellCept™ and Prograf™). After consuming an herbal remedy, consisting of a combination of *Agropyron repens* rhizome and *U. dioica* seed extracts (1:3, 5 mL, three times a day), the serum creatinine started to decline. After 46 days, U. *dioica* seed extract was used as a monotherapy for 3 months. Serum creatinine levels were normalized to acceptable levels [251].

##### Active Compounds and Possible Mechanisms of Efficacy

A variety of phytochemicals, including phenylpropanoids, flavonoids (such as chlorogenic acid, rutin and isoquercitrin, quercetin-3-O-rutinoside, kaempherol-3-O-rutinoside, and isorhamnetin3-O-glucoside), lignans (such as secoisolariciresinol), and coumarin (such as scopoletin), have been reported from nettle extracts. These extracts have shown anti-inflammatory and immunomodulatory effects with different selectivity toward the COX and LOX branches of the eicosanoid pathway [294,295]. Moreover, several plant sterols, such as sitosterol and its derivatives, have also been reported from the nettle root extract [296]. The root and leave extracts of *U. dioica* have shown immunomodulatory effects through different mechanisms, including lowering thromboxane production in human platelets and inhibiting the 12-LOX pathway. Different parts of the plant have shown antioxidant effects [294,297].

In a study, the effect of long-term injection of the Vβ8.3-specific superantigenic lectin *U. dioica agglutinin* (UDA) was investigated in MRL lpr/lpr mice (7 weeks of age). In contrast to the control group, injection of UDA (100 µg every two weeks for 4.5 months), inhibited the development of overt clinical signs of lupus and nephritis. Pathogenic T cell clones are thus found among the V8.3+ T cell population, which also contains an enlarged T cell clone. UDA affected autoantibody production in a sex-dependent manner [252].

##### Toxicity and Side Effects

Sweating and gastric discomfort are some of the side effects that have been reported to be associated with using stinging nettle. It should be noted that touching stinging nettle typically can cause skin irritation. Patients with renal conditions have been documented to experience hypersensitivity following consumption of this plant. Additionally, this plant has been shown to improve the effects of CNS depressive drugs. Consumption of stinging nettle concurrently with sedatives, such as lorazepam, phenobarbital, clonazepam, zolpidem, and others, may cause drowsiness and sleepiness [298].

#### 3.4.5. *Nelumbo nucifera* Gaertn.

*Nelumbo nucifera* belongs to the Nelumbonaceae family and is also known as the sacred lotus and water lily. In addition to being used as a vegetable and food, this plant has had many therapeutic uses from the past to the present. All parts of the plants (fruits, leaves, flowers, seeds, roots, rhizomes, buds, stems, anthers, stalks, plumules, and stamens) have been used in traditional medicine. The plant has been comprehensively studied to investigate its medicinal benefits such as anti-obesity, anti-diabetic, antioxidant, anti-amnesic, anti-thrombotic, anticarcinogenic, anti-inflammatory, immunomodulatory activity, anti-neurodegenerative, antiproliferation, cardiovascular activity, etc.

The effectiveness of lotus on SLE is due to the presence of procyanadins, polyphenols, and polysaccharides in its seeds [299].

The effectiveness of *N. nucifera* on SLE has been investigated in a study. In this study, 12-week-old MRL/MpJ-lpr/lpr mice were used. *N. nucifera* seeds extracted with ethanol contain (S)-armepavine (C_19_H_23_O_3_N). One group was given corn oil orally as a control; another group was given 5 and 10 mg/kg/day of oral (S)-armepavine; and the other group was given 20 mg/kg/day of oral cyclosporine. Mice were treated for 6 weeks. Finally, it was observed that taking (S)-Armepavine increased the life expectancy of mice and inhibited splenocytes proliferation and prevented lymphadenopathy. It also inhibited the expression of IL-2, IL-4, IL-10, and IFN-γ genes and inhibited T cells proliferation. Consumption of (S)-armepavine reduced proteinuria and anti-dsDNA autoantibody [94].

##### Toxicity and Side Effects

Despite the *N. nucifera*’s long history of therapeutic use, research on its possible toxicity and safety is required. Numerous investigations up to this point have partially supported the safety of *N. nucifera* [300]. The toxicity and safety profile of *N. nucifera* and its components have been examined in several research. A number of in vitro studies have been conducted to evaluate the toxicity of *N. nucifera* using normal cell lines. *N. nucifera* has not been found to significantly affect cell viability or to have any toxic effects, according to the findings of a number of in vitro investigations. The safety profile of *N. nucifera* has also been examined in numerous in vivo studies. In general, side effects from in vivo investigations include an increase in lymphocytes, a decrease in basophils, and a drop in creatinine, cholesterol, and hematocrit [301].

#### 3.4.6. *Artemisia annua* Pall.

*Artemisia annua* belongs to the Asteraceae family. *A. annua* has not only been used in TCM for various ailments but has also been identified as a medicinal plant in the United States, Europe, Australia, and Asia. This plant used to be found in western Asia and southeastern Europe. Today, it has spread all over the world and can be found in Australia, North and South America, and many parts of Asia and Europe. Since this plant is found in different parts of the world, it is known by many names, such as annual wormwood, sweet wormwood, Chinese wormwood, sweet sagewort, and sweet Annie. Antitumor, analgesic, anti-inflammatory, and antioxidant effects of *A. annua* have been discovered in studies on this species. In traditional medicine, this plant has been used in viral and bacterial diseases, jaundice, and autoimmune diseases such as rheumatoid arthritis and SLE, bacterial dysentery, hemorrhoids, and wound healing. It has also been used as an antipyretic in the treatment of tuberculosis and malaria. The 2015 Nobel Prize in Medicine was awarded for the discovery of sesquiterpene lactone artemisinin and its effects on the treatment of malaria [302]. Recently, the possibility of *A. annua* being useful in the treatment of COVID-19 has been investigated and satisfactory results have been reported [303,304,305].

##### Clinical Trial

In a clinical trial performed on 73 patients, including 36 patients with SLE and 37 patients with DLE, 60 or 80 mg of dihydroartemisinin was given orally for 9 weeks. Finally, dihydroartemisinin has been shown to be effective in most patients, and no serious side effects have been reported [306].

##### Phytochemicals and Possible Mechanisms of Action

The components identified from different parts of *A. annua* are classified as sesquiterpene lactones, coumarins, saponins, flavonoids, tannins, essential oils, polyalkenes, phenolic acids, fatty acids, proteins, and phytosterols [140,307,308]. Artemisinin, a sesquiterpene lactone in glandular hairs on the leaves and flowers of *A. annua*, is one of the important components of this plant, which is assumed to be one of the potential compounds against lupus. Apart from *A. annua*, other plants containing artemisinin, such as *Artemisia apiacea*, can also be effective in treating lupus [309].

Several semi-synthetic derivatives of artemisinin such as dihydroartemisinin, artemether, arteether, and artesunate have been investigated [140,302]. The effects of dihydroarteannuin (also known as dihydroqinghaosu, artenimol, or DHA) is one of the semi-synthetic derivatives of artemisinin. In a study, the effect of dihydroarteannuin and its mechanism on lupus in BXSB mice has been investigated. In this study, male BXSB mice were given dihydroarteannuin daily for ten days. One group was considered a control and the other three groups were given doses of 5 mg/kg, 25 mg/kg, or 125 mg/kg. As a result, dihydroarteannuin consumption was found to reduce TNF-alpha production, as well as nuclear factor-κB (NF-κB) activation and p65 subunit expression. Dihydroarteannuin inhibits NF-κB translocation to the nucleus and also inhibits IκB-α protein degradation [104]. In general, the level of TNF-alpha in the serum of SLE patients is higher than normal people. In addition, the expression of TNF-alpha receptors in peripheral blood lymphocytes of SLE patients is higher. NF-κB is a transcription factor located in the inactive cytoplasmic complex. It has two subunits, p50 and p65, and is attached to the IκB family, which are inhibitory proteins. Stimulation separates NF-κB from IκB and eventually translocates NF-κB to the nucleus, where it binds to DNA, inducing the expression of genes involved in the pathology of SLE progression [104].

In a study, the effect of *A. annua* was studied in female ICR mice, and it was found that, due to its suppressive effect on the immune system, it can be effective in diseases such as SLE and rheumatoid arthritis. Splenocytes of immunized mice were isolated and exposed to different concentrations, and finally, the number of specific antibodies was counted by indirect ELISA. The results indicated that the levels of a series of antibodies decreased following the consumption of ethanolic extract of *A. annua*. It was suggested that the plant might be useful in autoimmune diseases such as SLE and rheumatoid arthritis [228].

SM934 is another derivative of artemisinin that has more water solubility, bioavailability, and bioactivity but less toxicity. A study on MRL/lpr mice showed improvement in lupus syndrome. SM934 reduced the production of IFNγ and IL-17 by polyclonal CD4^+^ T cells activated by T cell receptor rearrangement in vitro, as well as the development of naive CD4^+^ T cells into Th1 and Th17 cells, but not Treg cells. In the in vivo study, administration of SM934 to mice for 4 weeks attenuated the renal lesion severity, proteinuria, and anti-dsDNA autoantibody. It also decreased the spleen size and the levels of serum IFN-Y and blood urea nitrogen. Following 8 weeks of consumption, the lifespan of mice increased. Ex vivo studies have shown an inhibition in the production of Th1 and Th17 while the level of Treg cells increased. In splenocytes, SM934 inhibited the complete activation of STAT-1, STAT-3, and STAT-5 proteins [97].

The effect of oral dihydroartemisinin (2 mg, daily, three days post-infection) on the immune system of BALB/C female mice infected with *Toxoplasma gondii* or *Plasmodium berghei* has been evaluated. Following the consumption of dihydroartemisinin, the number of B cells in the bloodstream and spleen cells decreased, and the ratio of T helper to CD8^+^ T cells increased. Dihydroartemisinin also decreased the proinflammatory cytokines. It is assumed that due to immunomodulating properties, dihydroartemisinin might be useful in autoimmune diseases such as SLE [105].

One of the factors associated with the progression of SLE is the imbalance between Treg/Th17. In a study, the effect of dihydroartemisinin alone or in combination with prednisolone on Treg/Th17 balance has been investigated. For this purpose, female BALB/c mice have been used. One group was considered the control, and one group was the SLE model group. The third group was given 100 mg/kg of dihydroartemisinin, the fourth group was given 5 mg/kg prednisolone, and the last group was given a combination of prednisolone and dihydroartemisinin with the same previous doses. Dihydroartemisinin and prednisolone were administered daily and orally for two months. As a result, the Treg/Th17 balance was restored, and the inflammation was inhibited. The effect of dihydroartemisinin has also been studied in vitro by isolating mouse spleen lymphocytes and exposing them to dihydroartemisinin or a combination of dihydroartemisinin and prednisolone. As a result, it was observed that the levels of TGF-β, IL-17, and Foxp3 increased, while transcription of RORγt decreased. Th17 cell differentiation is inhibited, but Treg cell differentiation is induced. Finally, dihydroartemisinin has been shown to have a synergistic effect on prednisolone [106]. There is also further evidence and studies to investigate the effect of *A. annua* on lupus [310,311,312].

##### Toxicity and Side Effects

Refer to the section on toxicity and side effects of artemisinin in the present text.

#### 3.4.7. *Linum usitatissimum* L.

The plant belongs to the Linaceae family and is also known as flaxseed and linseed.

##### Clinical Trials

In a two-year nonplacebo-controlled crossover study, 23 patients suffering from lupus nephritis were divided into two groups. One group received ground flaxseed (30 g/day for one year), and the second group was considered the control. At the end of the one-year period, the two groups were switched after a 12-week period of washout. Fifteen volunteers remained until the end of the trial. Eventually, it was observed that the viscosity of serum and plasma lipids remained unchanged, but serum creatinine decreased. Although microalbumin decreased during both flaxseed consumption and control times, a further decrease was observed during flaxseed treatment. It was concluded that in lupus nephritis, flaxseed appears to be renoprotective, but this interpretation is hampered by underpowering due to poor adherence and possible Hawthorne effects [240].

In another study, the effect of flaxseed on lupus nephritis was investigated. Nine patients with lupus nephritis enrolled while eight of them accomplished the study. Patients consumed 15, 30, and 45 mg of flaxseed daily for four weeks, with a washout of five weeks between doses. Finally, it was observed that blood viscosity and LDL decreased significantly, and there was a further decrease following an increase of the dose. Inhibition of AF-induced platelet aggregation, a decrease in serum creatinine, a decrease in proteinuria, an increase in complement C3, and decreased expression of CD11b have been observed. It was concluded that a daily consumption of 30 mg of flaxseed is tolerable and useful for patients with lupus nephritis due to its anti-inflammatory effect and beneficial effects on the kidney and atherogenic mechanism [241].

##### Phytochemicals and Possible Mechanisms of Action

Flaxseed oil, or linseed oil, is rich in omega-3 fatty acids. It also contains linolenic acid, linoleic acid, secoisolariciresinol diglucoside (SDG), lignans, and cyclic peptides [313,314]. Flaxseed contains polysaccharides with immunomodulatory properties such as FP-1. In a study, this polysaccharide stimulated immune responses by inducing mRNA expression of TNF-α, NO, IL-6, and IL-12 in murine macrophages [315].

Polyphenols, polysaccharides, and lignans of flaxseed have been reported to have antioxidant and anti-angiogenic properties [316,317,318]. Flaxseed oil has been reported to possess anti-inflammatory and immunomodulatory properties [319,320]. Flaxseed oil is rich in phytosterols such as campesterol, brassicasterol, stigmasterol, β-sitosterol, and Δ5-avenasterol [321]. These phytosterols have been repeatedly reported as anti-inflammatory agents [322]. Adding flaxseed oil to the co-culture of 3T3-L1 adipocytes, RAW 264.7 macrophages, showed a dose-dependent shift in cytokines toward IL-4 but a decrease in TNF-α. In the in vivo model (C57bl/6 mice), oral flaxseed oil (4 weeks) increased IL-4 cytokine, serum anti-ova IgG1, and IgE levels. Anti-ova IgG2a, IgG2b, and IgG3 levels were also reduced [319]. Some polyunsaturated fatty acids (PUFA), such as α-linolenic acid, have a variety of biological activities including neural stem cell proliferative, anti-atherosclerotic, and anti-inflammatory effects [209,215,323]. They might exhibit cardiovascular protective properties and might be useful in preventing the progression of nephritis in patients with lupus [240]. The beneficial effect of flaxseed on the kidneys has been reported in a number of studies [324,325,326,327].

In general, flaxseed reduces inflammatory responses and lowers blood pressure and vascular disease due to its PUFA content, such as omega-3. Since blood pressure is a risk factor for chronic kidney disease and flaxseed has an anti-inflammatory effect on the kidneys, flaxseed improves kidney health [313].

##### Toxicity and Side Effects

The flaxseed contains certain chemicals that have been recognized as potentially harmful, including cyanogenic glycosides and linatine, even though no toxicity has been documented in clinical investigations with dietary supplementation of flaxseed. Intestinal β-glycosidase changes the glycoside into cyanohydrin, which subsequently breaks down into hydrogen cyanide. Acute cyanide poisoning from hydrogen cyanide could put the nervous and respiratory systems at risk. However, consuming 15–100 g of flaxseed has not been reported to cause any rise in plasma cyanide levels above the baseline. Theoretically, 1–2 tablespoons of flaxseed will result in the production of between 5 and 10 milligrams of hydrogen cyanide when consumed. Toxic effects are quite unlikely to result from this. It is crucial to note that the idea that dietary flaxseed is toxic due to any of these components has not been proven scientifically [328].

#### 3.4.8. *Rehmannia glutinosa* (Gaertn.) DC.

*Rehmanniae Radix* (Di Huang) is the root of *Rehmannia glutinosa* (Gaertn.) DC. and belongs to the Orobanchaceae family. *Rehmanniae Radix* has long been used in TCM for medicinal purposes. Studies on *Rehmanniae Radix* have been shown to have antioxidant and anti-inflammatory effects and can lower blood sugar, activate the autonomic nervous system, and improve cognitive function. It has been used for treating dermatitis, cervical cancer, and nephrotic hypertension and improving liver damage. The effect of Rehmanniae Radix on lupus has also been investigated [302,329].

##### Clinical Trial

In a clinical trial in 72 patients with lupus, the combination of *Radix Rehmanniae* and *Radix Astragali* with glucocorticoid drugs was investigated. Patients were divided into control and treated groups. The control group was given prednisolone and cyclophosphamide, and the treated group was given a combination of Radix Rehmanniae and Radix Astragali on the basis of the control group. The duration of treatment in both groups was 6 months. The dose of prednisolone was reduced following the improvement in the patient’s condition. The dose reduction of prednisolone was greater in the treated group. In the treated group, there were fewer patients who had to increase the dose of prednisolone due to an aggravation of the disease. Infection, cardiovascular anomalies, hot flushes, insomnia, and Cushing’s syndrome were less common in the treated group. Although there was no difference in blood immunoglobulin G and blood complement 3 in the two groups, the protein in the 24 h urine was lower in the treated group. Therefore, following the use of *Radix Rehmanniae* and *Radix Astragal* with conventional Western medicine, fewer side effects were observed, and it was more convenient to withdraw corticosteroids [246]. A similar clinical trial was performed for patients with lupus nephritis, and it can be said that similar results were seen in general [330].

##### Phytochemicals and Possible Mechanisms of Action

In an in vitro study, the effect of fresh Rehmanniae radix Methanol extract has been investigated in adult female BALB/c mice. Therefore, mouse splenocytes were used. Finally, it has been observed that inflammatory cytokines such as IL-2, IFN-γ, IL-6 and IL-10 are reduced in the mouse splenocytes [247].

More than 100 chemical compounds have been reported from *R. glutinosa* that are mostly classified as iridoids, ionones, phenylethanoid glycosides, lignans, polysaccharides, and phenylpropanoids [331]. The polysaccharides of this plant have been reported to exhibit anti-inflammatory activity through suppression of IL-6 and TGFβ production on bacterial LPS-induced macrophages.

The anti-inflammatory effect of RG-B9, a polysaccharide isolated after processing of Rehmanniae Radix, involved AKT/ERK/JNK signaling pathway [332]. Two other polysaccharides, including SDH-WA and SDH-0.2A, have also been reported to have immunomodulatory properties. They increased lysozyme activity and TNF-α and IL-6 production by RAW264.7 cells. They did, however, reduce the secretion of lysozymes, TNF-α, IL-6, IL-1β, and nitric oxide by LPS-induced RAW264.7 cells [333]. In LPS-stimulated BV2 microglia, catalpol, an iridoid glucoside from *R. glutinosa* significantly suppressed LPS-induced secretion of proinflammatory mediators, NO and prostaglandin E2. Catalpol downregulated NO synthase and cyclooxygenase-2 expression. It also inhibited the TNF-α and IL-1β secretion. Moreover, catalpol downregulated the NF-κB signaling pathway, suppressed the expression of toll-like receptor 4 (TLR4), and lowered LPS-induced generation of ROS [334].

##### Toxicity and Side Effects

Although *R. glutinosa* is considered one of the safe plants, it can cause side effects such as dizziness, vertigo, headache, heart palpitations, nausea, diarrhea, allergies, and fatigue. It should be used with caution in patients with liver, digestive, and immune system problems [335].

#### 3.4.9. *Paeonia × suffruticosa* Andrews

*Paeonia suffruticosa* belongs to the family Paeoniaceae and is also known as mǔdān and Moutan Cortex. Due to its anti-inflammatory, antibacterial, sedative, anti-diabetic, and analgesic effects, it is used for inflammatory diseases, menstrual problems, cardiovascular diseases, and atherosclerosis. Many benefits of this plant are due to the large number of monoterpenoids glucosides in it [336]. The root bark of this plant has been used in traditional Chinese medicine for lupus nephritis [337].

##### Clinical Trials

The effect of Moutan Cortex on improving the condition of SLE patients in a clinical trial has been investigated. A total of 84 patients in the study group were given Moutan Cortex extract, and 84 patients in the control group were given common drugs. Finally, it was observed that following the use of Moutan Cortex extract, the percentage of Th17 cells decreased and the percentage of Th1 cells increased. Decreases were also observed in the IL-6 level, ESR, and SLEDAI scores. Compared to the control group, fewer side effects were observed with the use of Moutan Cortex. Therefore, taking Moutan Cortex improves the condition of SLE patients [245].

##### Toxicity and Side Effects

Moutan Cortex can be considered a safe raw material, and no research has been done to support its toxic effects. Although, benzoic acid, a component of the extracts that are thought to be toxic, is present in small amounts in this species. It is important to consider how easily heavy metals from soil, pesticides, air dust, irrigation water, vehicle and industrial exhaust gases, and fertilizers can contaminate raw materials. Exogenous elements like heavy metals, pesticide residues, or an excessive amount of sulfur from sulfur fumigation can contaminate Moutan Cortex. To assure the high quality of the raw material, it is crucial to determine the trace elements present in Moutan Cortex [338].

#### 3.4.10. *Paeonia lactiflora* Pall.

*Paeonia lactiflora* belongs to the family Ranunculaceae and the genus *Paeonia* and is also known as the Chinese peony, common garden peony, and shaoyao. *P. lactiflora* has a long history of use in TCM. Radix Paeoniae Alba and Radix Paeoniae Rubra are both derived from Paeonia roots, but in terms of the percentage of components and pharmacological actions, they are different. Radix Paeoniae Rubra, which is also known as chishao, RPR, and red peony root, is the dried root of *P. lactiflora* and has a cardiovascular and hepatoprotective effect. Radix Paeoniae Alba, which is known as baishao, RPA, and white peony root, is also the dried root of *P. lactiflora*, but there is also boiling and peeling in its production process. It affects the immune and nervous systems. Radix Paeoniae Alba and Radix Paeoniae Rubra both have antitumor and anti-inflammatory effects [339].

##### Clinical Trials

Total glycoside of paeony (TGP) in the hydroalcoholic extract of *Radix Paeoniae Alba*, which consists of more than 15 components. TGP has shown a direct anti-inflammatory effect by inhibiting the production of nitric oxide, leukotriene B4, and prostaglandin E2 [202].

A study investigated the effect of TGP in SLE patients. In this clinical study, one group of 29 cases received TGP for 5 years or more, the other group of 47 cases received TGP for one year or more (but less than 5 years), and the third group was the control. The results showed that following the daily intake of TGP, the required daily dose of prednisolone and cyclophosphamide decreased, and a decrease in SLEDAI score was observed, but no significant difference was observed in urinary protein. No side effects were observed following TGP use [115].

In another clinical trial, the effect of TGP on SLE patients was investigated on 70 SLE patients, who were divided into control and treatment groups. Both groups used conventional medicine, but the treatment group also received TGP for three months. Finally, it was found that the dose of glucocorticoids required by the patient decreased following the use of TGP. It was concluded that a combination of TGP and glucocorticoids might be beneficial for these patients. The side effects, including gastrointestinal effects, following the use of TGP were tolerable [116].

In a clinical trial, the effect of TGP on lupus nephritis was investigated. Forty patients were in the control group and were treated with prednisolone and cyclophosphamide. Forty patients were in the treatment group and were treated with TGP in addition to prednisolone and cyclophosphamide. As a result, following TGP intake, IL-18 and IL-6 levels decreased more than in the control group, and anti-dsDNA and serum creatinine levels decreased. Albumin and complement C3 levels increased following TGP intake. A meta-analysis on TGP, concluded that TGP is more efficient and safer when used in combination with conventional treatments. It could reduce the disease activity of SLE and the incidence of adverse reactions. Moreover, TGP could improve other outcomes related to SLE disease activity, including complement proteins (C3 and C4), immunoglobulins (IgA, IgM and, IgG), ESR, CRP, 24 h urine protein, and recurrence rate [117].

##### Phytochemicals and Possible Mechanisms of Action

*P. lactiflora* has therapeutic applications in lupus due to its anti-inflammatory and immunomodulatory effects. It has been many years since the decoction of the root of *P. lactiflora* has been used to treat SLE, rheumatoid arthritis, etc.

In a study, the effect of 12 weeks of treatment with Radix Paeoniae Rubra on lupus nephritis was investigated. MRL/lpr lupus mice were divided into three groups: control, prednisolone, and Radix Paeoniae Rubra. The results showed a decrease in renal pathological damage and urinary protein levels. Also, the expression of intercellular cell adhesion molecule-1 (ICAM-1), vascular cell adhesion molecule-1 (VCAM-1), and platelet endothelial cell adhesion molecule-1 (PECAM-1) decreased. Moreover, following the use of Radix Paeoniae Rubra, the required dose of prednisolone was reduced [244].

In another study on female MRL/lpr mice, the effect of oral administration of 50 mg/kg/d of TGP was investigated. As a result, ERα expression decreased, and DNA methyltransferases (DNMTs) expression increased. Besides a decrease in renal damage and the expression of IFN-γ, IL6, and IL12 cytokines, the serum dsDNA levels were inhibited [118]. In another study, following the consumption of TGP by MRL/lpr mice, the urinary protein content and the levels of anti-dsDNA antibodies and antinuclear antibodies (ANA) decreased [119].

In an in vitro study, the effect of TGP was investigated on the expression and DNA methylation status of the ITGAL gene (CD11a) in CD4^+^ T cells isolated from patients with SLE. TGP led to down-regulation of ITGAL mRNA and protein levels. In addition, DNA methylation of the ITGAL promoter was increased, which can result in the repression of the CD11a gene expression [120].

Among the components of TGP, paeoniflorin (Pae), hydroxyl-paeoniflorin, paeonin, albiflorin, and benzoylpaeoniflorin can be named. Paeoniflorin, accounting for about 40% of TGP, is the major active component of TGP. This compound has been shown to modify immune cell activities, reduce inflammatory medium formation, and correct aberrant signal pathways. Paeoniflorin has been found to regulate a variety of signaling pathways, including GPCR pathway, MAPKs/NF-κB pathway, PI3K/Akt/mTOR pathway, JAK2/STAT3 pathway, TGFβ/Smads, etc. [201].

##### Toxicity and Side Effects

Although there is not a lot of information about *P. lactiflora*’s toxicity, peonies are typically regarded as non-toxic in texts on traditional medicine. According to the research, the roots of paeony species are not toxic, but elevated doses of some of their components, such as pyrethrin I and phenol, can be harmful [340]. In the section of this article on purified molecules from natural sources, the side effects of this plant and TGP compound have been discussed.

#### 3.4.11. *Scutellaria baicalensis* Georgi

*Scutellaria baicalensis* belongs to the family Lamiaceae and is also known as Baikal skullcap or Chinese skullcap. Wogonin, baicalin, and baicalein are some of the components of *S. baicalensis*. [248] Baicalin is a flavonoid isolated from the root of *S. baicalensis* and its anti-inflammatory, anti-cancer, and antioxidant effects have been studied [99].

##### Phytochemicals and Possible Mechanisms of Action

An in vitro study on pristane-induced lupus BALB/c mice found that the use of *S. baicalensis* downregulated the production of proinflammatory cytokines such as TNF-α, IL-6, IL-10, and IFN-γ. Also, the expression of CD69^+^ CD4^+^ T cells and CD4^+^ T cells decreased, but CD8^+^ did not decrease [248].

In another in vivo study, BALB/c mice were divided into three groups: healthy control mice, lupus control mice, and baicalin-treated mice. Mice were treated with 50 mg/kg of baicalin for 10 days. Finally, it was shown that following the use of baicalin, abnormal activation of T cells was downregulated and overproduction of IL-6 and PGE2 was inhibited [99,160].

To evaluate the effect of baicalin, a study was performed on lupus-prone MRL/lpr mice in which 200 mg/kg of baicalin was used peritoneally daily for 4 weeks. It was observed that following the use of baicalin, urine protein decreased, anti-dsDNA antibody titers were inhibited, and lupus nephritis was attenuated. Baicalin inhibits IL-21 production and Tfh cell differentiation and induces Foxp3^+^ regulatory T cell differentiation [98].

##### Toxicity and Side Effects

Refer to the section on toxicity and side effects of baicalin in this text.

#### 3.4.12. *Gentiana macrophylla* Pall.

*Gentiana macrophylla* belongs to the Gentianaceae family, which is also known as Qinjiao. The Gentiana genus has protective effects on the cardiovascular, gastrointestinal, and reproductive systems. They have long been recognized for their immunomodulatory properties, as well as their beneficial effects on the skin, liver, and joints. *Gentiana* spp. have been used to control visceral pain [65,66,341]. *Gentiana macrophylla* root has been used in TCM to treat inflammation and pain and systemic lupus erythematosus [239].

##### Clinical Trial

In a clinical trial, the effect of *G. macrophylla* was compared with that of prednisolone. Sixty-two patients with SLE were treated with *G. macrophylla* complex tablets in the form of 10 tablets twice a day or 5 tablets three times a day with 10 to 30 mg of prednisolone daily. In the control group, 19 SLE patients were treated with prednisolone alone. As a result, it was observed that the recovery rate was significantly higher in the Gentiana group. Improvement of nephropathy, erythema, and arthralgia and restoration of ESR, LE cells, C3, and CH50 following *G. macrophylla* consumption was higher in than the control group, and no significant complication was observed [238].

##### Animal Studies

In a study, the cardiac protective effect of *G. macrophylla* on lupus mice was investigated. Thirty female NZB/W F1 mice were used, which were divided into three groups that included the control group, the cholesterol-consuming group, and the cholesterol- and *G. macrophylla*-consuming group. After 12 weeks, the mice’s heart tissue was used for further experiments. The results showed that following the use of *G. macrophylla*, cholesterol-aggravated apoptosis decreased, IGF-1 survival signal increased, and anti-apoptotic proteins increased. Therefore, *G. macrophylla* protects the heart against cholesterol-aggravated apoptosis in mice with lupus, so it can be useful in the treatment of CVD in patients with SLE [67].

In another study, the hepatoprotective effect of *G. macrophylla* on lupus mice was investigated. The female NZB/W F1 mice were divided into four groups and participated in the experiment for 8 weeks. The livers of mice were used for further experiments. Finally, they found that taking *G. macrophylla* could be effective in reducing liver inflammation in SLE patients [239].

##### Toxicity and Side Effects

In vivo studies on the Gentiana genus have not revealed any significant toxic effects when given elevated doses [65]. In Kunming mice treated with 500 mg/kg of *G. macrophylla* root extract, no abnormal performance or mortality was observed [342].

#### 3.4.13. *Glycyrrhiza glabra* L.

*Glycyrrhiza glabra* belongs to the Fabaceae family and is also known as liquorice, sweet wood, and mulaithi [343]. *G. glabra* root contains glycyrrhizin and its derivatives, which are in the class of saponins [344]. *G. glabra* has long been used to treat a wide range of diseases. Studies have shown that it has anti-inflammatory, antioxidant, immunostimulatory, anticoagulant, hepatoprotective, and neuroprotective properties, as well as antibacterial, antiviral, antifungal, and anti-malarial effects [69,345,346].

##### Phytochemicals and Possible Mechanisms of Action

Licorice root is rich in triterpenoids and flavonoids, which are known for their antioxidant and anti-inflammatory properties, mostly by decreasing TNF, MMPs, PGE2, and free radicals [347].

*G. glabra* has been reported to be beneficial in improving SLE. High mobility group box 1 (HMGB1) has proinflammatory effects and an immune-stimulatory function and plays a role in the pathogenesis of inflammatory and autoimmune diseases such as SLE. Glycyrrhizin has the blocking effect of HMGB1 [110,348]. Elevated HMGB1 levels have been shown to be associated with exacerbation of SLE and elevated levels of proinflammatory cytokines such as IL-6 and TNF-*α*. The results of an animal study on female BALB/c mice that received glycyrrhizin (0.5 mg/day for two months) demonstrated that glycyrrhizin’s inhibition of HMGB1 function caused a sharp decline in serum HMGB1 levels, which in turn decreased the severity of SLE [110]. Glycyrrhizin directly binds to HMGB1, reducing both the extracellular release of HMGB1 and its cytokine actions [348].

In another study based on HMGB1, glycyrrhizin was used as an HMGB1 blocker. Glycyrrhizin (10 mg/kg) was injected every other three days into female BALB/c mice for 12 weeks. Finally, an improvement in lupus nephritis was achieved following the use of glycyrrhizin. Decreased levels of anti-dsDNA antibodies and decreased levels of inflammatory cytokines, as well as decreased glomerular IgG and C3 deposition and reduction of proteinuria, were observed [349].

In an in vitro study, glycyrrhizin was shown to inhibit the immunocomplex formation of 60S acidic ribosomal P proteins from porcine liver when combined with patient serum for SLE. It was concluded that a relatively high dose of glycyrrhizin could prevent the immunocomplex formation of 60S acidic ribosomal P proteins with their specific antibodies in the sera of SLE patients [112].

No clinical trial was found on the efficacy of licorice on SLE. Considering the results of preclinical studies and the application of licorice as an anti-inflammatory agent in a variety of diseases, further studies might lead researchers to find effective remedies.

##### Toxicity and Side Effects

The most significant adverse effects of glycyrrhizin and licorice are secondary diseases brought on by hypokalemia and hypertension. Additionally, it may result in fatal arrhythmias and cardiomyopathy. Hypokalemia, hypertension, anorexia nervosa, prolonged gastrointestinal, advanced age, and being of female sex all enhance the risk of adverse consequences from licorice. It should be mentioned that the positive effects of beta blockers and angiotensin-converting enzyme inhibitors (ACEIs) can be countered by the hypertensive impact of licorice [350,351].

#### 3.4.14. *Antrodia camphorata*

*Antrodia camphorata* is a fungal parasite on *Cinnamomum kanehirai.* It is also known as “stout camphor fungus”. *Antrodia camphorata* has long been used in traditional medicine in China and Taiwan and is used to treat a wide range of diseases. Crude extracts of *A. camphorata* have been shown to have anti-cancer, antioxidant, anti-inflammatory, immunomodulatory, hepatoprotective, neuroprotective, anti-hypertensive, and vasorelaxant effects [153].

A study investigated the effect of *A. camphorata* on nephritis in SLE-prone NZB/W F1 mice. For this purpose, for 12 weeks, 100, 200, and 400 mg/kg of *A. camphorata* extract were administered orally on 5 consecutive days per week. As a result, the kidney glomerular basement membrane’s thickness was reduced and urine protein and serum BUN levels were markedly controlled by the extract of *A. camphorate* (400 mg/kg) [46]. Antroquinonol was reported to be the main active ingredient (refer to the antroquinonol section in this text).

##### Toxicity and Side Effects

Refer to the section on toxicity and side effects of antroquinonol in this text.

#### 3.4.15. *Astragalus propinquus* Schischkin (syn. *Astragalus membranaceus* (Fisch.) Bunge)

*Astragalus membranaceus* belongs to the Fabaceae family and is also known as Mongolian milkvetch. It consists of the components astragaloside, astragalus flavonoids, and astragalus polysaccharide. It has an anti-inflammatory effect and reduces proteinuria and creatinine. It has been used to treat kidney disease [352]. *A. membranaceus* is used in lupus nephritis and its effects have been reviewed in articles. In a study, the effect of *A. membranaceus* and *Tripterygium hypoglancum* on SLE patients was investigated, and it was observed that NK activity decreased and therefore disease activity decreased [229]. A bioinformatics study has attempted to investigate the mechanism of the effect of *A. membranaceus* on lupus nephritis [352].

##### Toxicity and Side Effects

*A. membranaceus* has an LD_50_ of about 40 g/kg, which makes it safe and non-toxic. However, it has been found through in vivo research that a dose of 1 mg/kg of this plant in the form of AS-IV can have side effects such as fetal toxicity and reproductive toxicity. As a result, it should be used carefully throughout pregnancy and the postpartum period. However, astragalus extract is generally safe and has no significant side effects [353].

#### 3.4.16. *Bryophyllum pinnatum* (Lam.) Oken

*Bryophyllum pinnatum* belongs to the Crassulaceae family and is also known as Kalanchoe pinnata, air plant, Zakham-e-hyat, life plant, and cathedral bells. The constituents of *B. pinnatum* include alkaloids, glycosides, triterpenes, cardienolides, flavonoids, steroids, lipids, and buffadienolides. It is used for a wide range of diseases and has been found to have antibacterial, antileishmaniasis, and antimutagenic effects, as well as hepatoprotective and nephroprotective effects. *B. Pinnatum* can be beneficial in the treatment of SLE due to its anti-inflammatory and immunosuppressive effects [354]. The effect of *B. pinnatum* on SLE has been investigated.

In a study on BALB/c mice, mice were treated with different doses of ethanolic extract of *B. pinnatum* leaves for 12 weeks. For this purpose, 4 groups including control and those with doses of 10.5, 21, and 42 mg/kg/day were used. As a result, following the consumption of *B. pinnatum*, a decrease in TNF-α, IL-17, IL-12, CRP, and matured B cells was observed. On the other hand, there was an increase in complement C3 and C4 and TGF-β. No specific side effects were reported with *B. pinnatum* [230,231].

In an in vitro study performed with the help of spleen cells of BALB/c mice, the effect of ethanolic extract of *B. pinnatum* leaves at doses of 0, 0.02, 0.1, or 0.5 µg/mL was investigated. As a result, *B. pinnatum* reduces B cell maturation and increases B cell apoptosis and decreases NF-ĸB p65 expression [232]. The effect of *B. pinnatum* on B cells has also been investigated in silico [355,356].

In vivo, the effect of aqueous extract of *B. pinnatum* leaf on lupus nephritis in female Balb/c mice was performed. Mice in the treatment group received 200, 400, or 600 mg/kg/day aqueous extract of *B. pinnatum* orally for 21 days. Eventually, it was found that proteinuria levels and glomerular inflammation were reduced. On the other hand, with the help of in silico studies, an attempt has been made to find the flavonoid composition that binds to the glucocorticoid receptor. It has been shown that bryophyllin A is probably the active compound of *B. pinnatum* that has an anti-inflammatory effect [233]. There is also further evidence and studies that investigate the effect of *B. pinnatum* on SLE and lupus nephritis [357,358,359,360].

##### Toxicity and Side Effects

Toxicological tests have been carried out mostly on leaf extracts to determine the safety of *B. pinnatum*. Even though the majority of research works have demonstrated low toxicity and acceptable safety, some have noted its cytotoxicity. For this plant, abnormalities in the animal’s testicles have also been observed. As a result, it’s seemed that *B. pinnatum* can be used safely in acute situations, but further research is required to determine its chronic toxicity [361].

#### 3.4.17. *Anemarrhenae aspheloidis*

*Anemarrhenae aspheloidis* belongs to the family Asparagaceae and is also known as Zhi Mu. Anemarrhenae rhizoma has been used in TCM for many years to treat various ailments. One of the compounds of anemarrhenae rhizoma is mangiferin, which has antioxidant, anti-inflammatory, and immunomodulatory effects. The effect of mangiferin extracted from anemarrhenae rhizomaand *Mangifera indica* on lupus nephritis has been investigated (see the section on mangiferin in this text) [362].

##### Toxicity and Side Effects

Refer to the section on toxicity and side effects of mangiferin in this text.

#### 3.4.18. *Camellia sinensis* (L.) Kuntze

*Camellia sinensis* belongs to the family Theaceae and is generally known as tea. The compounds in *C. sinensis* depend on various factors such as geographical environment, growing season, etc., but generally contain flavanols, flavonols, polyphenolic acids, and flavonol glycosides. *C. sinensis* has anti-inflammatory, antioxidant, and anti-arthritis effects [363]. The effect of *C. sinensis* on lupus has been studied. In an in silco study, the potency of green tea phytoconstituents as immunomodulators, anti-apoptosis agents, and anti-pyroptosis agents in SLE was investigated. The result of molecular docking can explain the mechanism of the active compound as anti-apoptosis and anti-pyroptosis. The docking results suggested theaflavin as one of the most active constituents [364].

##### Clinical Trial

The effect of *C. sinensis* on the improvement of SLE has been investigated due to its anti-inflammatory and immunomodulatory effects. In this clinical trial, 68 SLE patients were divided into control and study groups. The study group was treated with 1000 mg of *C. sinensis* extract daily for 12 weeks. Finally, it was observed that following the consumption of *C. sinensis*, the quality of life of patients and their general health increased and their disease activity decreased [234].

##### Toxicity and Side Effects

Although *C. sinensis* is one of the most commonly used and safe plants, a number of side effects have been reported for its excessive consumption, including diuresis, tremors, irritability during the day, heart irregularities, nervousness, anxiety, headache, and hypotension. In patients with anxiety, poor cardiovascular systems, renal disorders, and hyperthyroidism, it has been suggested that tea consumption be restricted [365].

#### 3.4.19. *Curcuma longa* L.

*Curcuma longa* belongs to the Zingiberaceae family and is also known as turmeric. *C. longa* is widely used in Asian traditional medicine due to its medicinal properties. Since curcumin can interact with different molecular and cellular targets, it exhibits a wide range of pharmacological effects including anti-inflammatory, antioxidant, antimicrobial and chemotherapeutic activity [21]. It also has hepatoprotective effects and is useful in gastrointestinal disorders [366]. The effect of *C. longa* on lupus has been studied. One of the most active constituents is curcumin (see the section on curcumin in this text).

##### Clinical Trials

In a clinical trial, the effect of turmeric on the improvement of lupus nephritis has been investigated. For this purpose, 24 patients with lupus nephritis were divided into study and control groups. The study group was treated with 1500 mg of turmeric daily for 3 months. As a result, following turmeric consumption, proteinuria and hematuria decreased, and systolic blood pressure also decreased. No side effects were observed following short-term use of turmeric in this clinical trial [235].

##### Toxicity and Side Effects

Refer to the section on toxicity and side effects of curcumin in this text.

### 3.5. Other Plants with Lower Evidence for Lupus Conditions

In addition to the herbs listed so far, other herbs that have anti-inflammatory, antioxidant, or immunomodulatory effects can help improve SLE. By using these herbs with conventional treatment, the patient might experience fewer adverse effects, and a lower dosage of conventional medicine might be needed.

*Cinchona officinalis L.* belongs to the family Rubiaceae and is known as cinchona and Peruvian bark. Important components in *C. officinalis* include alkaloids such as Quinine, Quinidine, Chichonine, and Cinchonidine. It has anti-malarial, anti-inflammatory, antioxidant, antimicrobial, and anti-cancer effects and is used to treat lupus, malaria, etc. [367,368]. The plant and its phytoconstituents were applied to treat COVID-19 due to its anti-inflammatory and immunomodulatory properties [369,370].

*Bupleurum falcatum* L. (Apiaceae) is known as Chinese thoroughwax and sickle-leaf hare’s ear. Saikosaponins extracted from *B. falcatum* have anti-inflammatory, immunoregulatory, and anti-cancer effects. Investigating the potency of Saikosaponins to attenuate symptoms in autoimmune diseases such as lupus can be beneficial [371].

*Centella asiatica(L.) Urb.*(Apiceae family) is known as *Hydrocotyle asiatica*, *Indischer Wassernabel*, and Indian pennywort. [372] It contains triterpene saponins, madecassic acid, asiatic acid, asiaticoside, and madecassoside. It is mostly used to heal wounds, but it has also been used for skin conditions caused by lupus, leprosy, eczema, psoriasis, varicose ulcers, etc. [373].

*Tinospora sinensis (Lour.) Merr. *(syn. *Tinospora cordifolia*) belongs to the family Menispermaceae and is also known as guduchi, heart-leaved moonseed, and gurjo. This plant can be used in SLE due to its immunomodulatory effect. It has been reported for its anti-inflammatory, immunomodulatory, antioxidant, antibacterial, antifungal, and anti-diabetic effects. It has been used in some inflammatory and autoimmune diseases such as SLE, rheumatoid arthritis, psoriasis, etc. [374].

*Acacia farnesiana* (L.) Willd. (Fabaceae) is known as sweet acacia, needle bush, and huisache. *A. farnesiana*, due to its proteins, lectin, and α-amyrin, β-amyrin, and lupeol have anti-inflammatory and antioxidant effects and can down-regulate proinflammatory mediators [375,376,377].

*Morinda citrifolia* L. belongs to the family Rubiaceae and is also known as noni. In addition to its nutritional value, *M. citrifolia* has several biological activities, including, antibacterial, anti-fungal, anti-inflammatory, antioxidant, and antituberculosis effects, which have been reported for this plant and its phytochemicals [378]. *M. citrifolia* has also been claimed to be beneficial in treating lupus [379,380].

*Cornus officinalis* Siebold and Zucc. belongs to the Cornaceae family and is known as Asiatic Dogwood, Japanese Cornel Dogwood, and Shan Zhu Yu. Its ripe and dried fruit is called Corni Fructus. Corni Fructus has been used in traditional Chinese medicine (TCM) for a variety of conditions. It has exhibited antioxidant, anti-inflammatory, nephroprotective, hepatoprotective, neuroprotective, hypoglycemic, and anti-cancer effects [381]. The combination of Corni Fructus with other plants of traditional Chinese medicine has been used to treat lupus, although we could not find a study that has specifically examined the effect of Corni Fructus on SLE. It has been assumed that, owing to its anti-inflammatory and nephroprotective effects, it can be beneficial for patients with lupus. There are studies on the mechanism of its anti-inflammatory and kidney protective effect [382,383,384].

*Allium sativum* L. (Alliaceae) is known as garlic. Alliin and allicin are the most important sulfur components in *A. sativum*. It has exhibited a wide range of therapeutic properties, including anti-inflammatory, antioxidant, antimicrobial, antifungal, immunomodulatory, antiatherosclerotic, anti-hypertensive effects, etc. [385] *A. sativum* has also been suggested to be useful in improving lupus patients [386].

*Wolfiporia extensa*, or *Poria cocos*, is a fungus that belongs to the family Polyporaceae and grows on the roots of the pine tree. *Poria cocos* has long been used extensively in traditional Chinese medicine and is also known as Fuling, poria, and hoelen. Due to the presence of triterpenoid and ergosterol compounds, it has anti-inflammatory effects and also affects the immune system, so it is used in diseases such as rheumatoid arthritis and SLE. In addition to anti-inflammation and immunomodulation effects, this fungus also has antitumor, anti-diabetic, anti-aging, and antioxidant effects [387,388,389].

Evening primrose oil (EPO) can also be helpful in the healing process of lupus and is obtained from the seeds of *Oenothera biennis* L., which belongs to the Onagraceae family. The plant is also known as evening primrose, evening star, and sundrop. Evening primrose oil (EPO) contains a high concentration of γ-linolenic acid (GLA), which has anti-inflammatory, antioxidant, radical scavenging, and immunomodulatory properties [390,391]. Some clinical trials have shown that EPO can be beneficial in improving some inflammatory conditions such as atopic eczema and atopic dermatitis. There are studies on the effect of EPO on arthritis [391,392]. Due to the presence of γ-linolenic acid and prostaglandin E1, its use can be effective in SLE patients.

*Andrographis paniculata* (Burm.f.) Nees belongs to the family Acanthaceae and is also known as green chiretta and creat and Chuan Xin Lian. The aerial parts of this plant have been used in traditional Chinese medicine to treat inflammation, pain, and detoxification. *A. paniculata* contains flavonoids, polyphenols, and diterpenoids [393]. Immunomodulatory effects have been reported for the aqueous extract of *A. paniculata* leaves on rats [394].

*Phyllanthus emblic* G.L.Webster belongs to the family Euphorbiaceae. The genus *Phyllanthus* has long been used extensively in immune-related diseases such as SLE and has been shown to have an immunomodulatory effect [395].

*Coriandrum sativum* L. (Apiaceae): The essential oils in the seeds and leaves of this plant, such as linalool, are responsible for many of the plant’s benefits. *C. sativum* has anti-inflammatory, antioxidant, antibacterial, anti-cancer, immunostimulatory effects, etc. [396]. Consumption of this plant could be effective in lupus patients due to its anti-inflammatory and immunomodulatory effects [30].

*Boswellia* spp. (Burseraceae): The four main species of Boswellia, *B. sacra*, *B. frereana*, *B. papyrifera*, and *B. serrata* produce frankincense (also known as olibanum). Among the chemical compounds of frankincense 3-O-acetyl-11-keto-β boswellic acid, α- and β-boswellic acids, 11-keto-β-boswellic acid and other boswellic acids, lupeolic acids, incensole, cembrenes, triterpenediol, tirucallic acids, and olibanumols can be named. Frankincense exhibits anti-inflammatory effects through a variety of mechanisms including inhibition of leukotriene synthesis, cyclooxygenase 1/2 and 5-lipoxygenase, and oxidative stress and by regulation of immune cells from the innate and acquired immune systems. Additionally, it modifies signal transduction, which is in charge of cell cycle arrest as well as the suppression of proliferation, angiogenesis, invasion, and metastasis [397]. The major components of frankincense are boswellic acids, among which the most important and abundant is 3-O-acetyl-11-keto-β-boswellic acid (AKBA). This compound is a strong inhibitor of 5-lipoxygenase with anti-inflammatory and anti-arthritic properties [398]. Another suggested mechanism for the anti-inflammatory effects of boswellic acid is potently inhibiting cathepsin G [399].

In clinical trials, frankincense and its phytochemicals were found to be effective in treating a range of inflammatory disorders, such as osteoarthritis, multiple sclerosis, asthma, psoriasis and erythematous dermatitis, plaque-induced gingivitis, and pain [397,400,401]. We could not find a clinical trial investigating the efficacy of frankincense on SLE, but due to its anti-inflammatory and immunomodulatory effects, it might be poetically effective.

*Dioscorea polystachya* Turcz. (syn. *Dioscorea batatas* Decne.) belongs to the Dioscoreaceae family and is also known as Chinese yam or cinnamon-vine. In addition to being used as food in China, it has also been used in traditional Chinese medicine to treat various diseases such as asthma, diabetes, etc. It has anti-inflammatory and antioxidant effects [402,403] and has also been used to treat lupus [402,403,404].

*Ocimum gratissimum* L. belongs to the Lamiaceae family and is also known as Ram Tulshi, clove basil, and African basil. In addition to being used in food in some countries, this plant is also used in the treatment of some diseases. *O. gratisimum* has anti-malarial, anti-inflammatory, and antioxidant effects [405]. Owing to its immunomodulatory and anti-inflammatory effects, it can be effective in treating SLE patients [30,406].

*Uncaria tomentosa* (Willd. ex Schult.) DC. belongs to the Rubiaceae family and is also known as cat’s claw. The roots and bark of this plant have been used in traditional medicine to treat various diseases such as inflammation, viral infections, urinary tract infections, asthma, etc. *U. tomentosa* has anti-inflammatory, antioxidant, antimicrobial, and immunomodulatory effects and can be effective in treating lupus [407,408]. There is a case report that an SLE patient has ended up with acute renal failure following daily use of *U. tomentosa* [409].

*Clerodendrum trichotomum* Thunb. (Verbenaceae) is known as Chou Wu Tong and Kusagi. *C. trichotomum* has antioxidant, anti-inflammatory, analgesic, and sedative effects [410,411]. Due to the presence of taraxerol, friedelin, lupeol, and betulinic acid, it has an immunomodulatory effect [30].

*Scrophularia ningpoensis* Hemsl. belongs to the family Scrophulariaceae and is also known as Xuanshen. In traditional Chinese medicine, it has been used for many years to treat various diseases. Among the components of *S. ningpoensis*, phenylpropanoid glycosides and iridoid glycosides can be named to possess anti-inflammatory effects. The plant has been used to treat liver disease, cardiovascular disease, and diabetes and has antioxidant, anti-inflammatory, and anticarcinogenic effects [412]. Its effectiveness in lupus has also been considered [413].

## 4. Conclusions

Overall, a variety of natural molecules and their derivatives, in purified and structurally elucidated form, have been reported to exhibit beneficial effects in lupus conditions. Among these molecules, artemisinin and its derivatives, antroquinonol, baicalin, curcumin, emodin, mangiferin, salvianolic acid A, triptolide, and the total glycosides of paeony (TGP) have potential to be considered for further drug development studies. They showed their efficacy through interaction with various immune mediators, cytokines, and transcription factors such as nuclear factor kappa B (NF-κB), inhibition of anti-dsDNA, etc. Additionally, some omega-6 and omega-3 PUFAs, such as EPA, DHA, α-linolenic acid, and γ-linolenic acid, have been shown to be beneficial by lowering anti-dsDNA, TNF-α, IL-1, IL-1, IL-2, and/or CRP levels.

Some minerals (calcium, iron, selenium, and zinc) and vitamins (vitamins A, B, C, D, and E) have shown potential to exhibit degrees of beneficial effects. Considering the reported clinical trials on medicinal plants and fungi, *T. wilfordii*, *O. sinensis*, *G. lucidum*, *A. annua*, *U. dioica*, *L. usitatissimum*, *R. glutinosa*, *P. × suffruticosa*, *P. lactiflora, G. macrophylla*, and *C. longa* have exhibited efficacy against lupus conditions. Due to the small number of clinical trials and the small number of patents in each trial, it is not possible to do a meta-analysis on any of the phytochemicals or herbal medicines listed above. However, since many of these herbal medicines, minerals, or vitamins have a history of human consumption, until more studies are done, the use of these products in the form of complementary products can be beneficial for patients.

## Figures and Tables

**Table 1:** Main botanical aspects and pharmacological benefits of medicinal plants, mushrooms, and fungi advised for lupus conditions.

Plant Name (Scientific Name or Species)	English Name	Endemic Region Endemic Name	Family	Main Parts Used	Main Botanical Characteristics (Description)	Preparations	Main Pharmacological Benefits/Activities/Properties	Reference
Taiwanofungus camphoratus (M.Zang and C.H.Su) Sheng H.Wu, Z.H.Yu, Y.C.Dai and C.H.Su (Basionym: *Ganoderma camphoratum*) M.Zang and C.H.Su	Stout camphor fungus	In Taiwan as “Niu-chang-chih” or “Chang-chih” or “Niu-chang-ku” or “Chang-ku”	Polyporaceae	Fruiting bodies	A parasitic fungus on the endemic rotting trees of *Cinnamomum kanehirai Hayata* causing a brown heart rot	Extract	Antioxidant Anti-inflammatory Immunomodulatory Antimicrobial Anti-diabetic Hepatoprotective Neuroprotective Vasorelaxation Antitumor Anti-hypertensive	[46,47,48]
*Artemisia annua* L.	Annual absinthe, Sweet wormwood, Sweet annie Annual mugwort, Annual wormwood, Sweet wormwood, Chinese wormwood, Sweet sagewort	-Mild climates of Asia -In China as Qinghao	Asteraceae	Leaves	An annual plant with hairless brown erect stems (a height of 50 to 100 cm) and tender leaves about 3 to 5 cm long and many small yellowish-green flowers and brown rounded seeds	Extract	Anti-inflammatory Immunomodulatory Immunosuppressive Antioxidant, Antitumor Anti-malarial, Antibacterial Antifungal, Anti-cancer Anti-obesity, Hepatoprotective, Anti-asthmatic	[49,50]
*Astragalus propinquus* Schischkin (Syn. *A. membranaceus* fo. *propinquus* (Schischk.)) Kitag.	Mongolian milkvetch	Huang Qi, in Chinese, Ogi, in Japanese, Milk-Vetch, in English, and Gavan, in Persian	Fabaceae (or Leguminosae)	Roots	A Perennial flowering plant 50–150 cm high, including a straight and long (up to 50 cm) cylindrical root, erect stems branched in the upper parts with small ovate-lanceolate or elliptical leaves	Extract	Anti-inflammatory Immune system boosting Antioxidant, Anti-cancer Neuroprotection, Renoprotection (reduces proteinuria and creatinine), Hepatoprotection, Hypoglycemic Anti-osteoporosis, Anti-fatigue, Cardioprotection	[51]
*Kalanchoe pinnata* (Lam.) Pers. Syn. *Bryophyllum pinnatum* (Lam.) Oken	Air plant Canterbury bells Cathedral bells Curtain plant Life-plant Fortune plant Good luck leaf Green mother of millions Air plant Mexican love plant	Madagascar In Brazil as “folha-da-fortuna” or “folha-de-pirarucu” or saião “coirama”, In Caribbean as “bruja”	Crassulaceae	Leaves	A perennial succulent herb 0.3–1.2 m tall, including four-angled stems and fleshy simple/compound dark green leaves to 20 cm long and red pendulous flowers	Extract	Anti-inflammatory, Antinociceptive Antianaphylactic Immunomodulator, Immunosuppressive, Antitumor, Antiulcer, Anti-diabetic, Hypotensive, CNS depressant, Antimicrobial Antileishmania, Anthelmintic, Gastroprotective Hepatoprotective Anti-urolithiatic	[52,53,54]
*Camellia sinensis* (L.) Kuntze	Tea plant tea shrub Tea tree	China and Southeast Asia	Theaceae	Leaves	Evergreen shrub or small tree with strong taproot and yellow-white flowers with seven or eight petals	Extract	Antioxidant, Analgesic Anti-inflammatory, Anti-cancer, Anti-fibrotic, Antimutagenic, Cholesterol lowering, Cardiovascular protection, Hepatoprotective, Neuroprotective Anti-diabetic, Anti-obesity Analgesic	[55,56,57]
*Curcuma longa* L.	Turmeric	Indian subcontinent South-east Asian Curcum in the Arab region Indian saffron Haridra (Sanskrit, Ayurvedic) Jianghuang (yellow ginger in Chinese) Kyoo or Ukon (Japanese)	Zingiberaceae	Rhizome	A perennial herbaceous plant with 1 m high with highly branched, yellow to orange, cylindrical, aromatic rhizomes and oblong, pointed leaves and bears funnel-shaped yellow flowers	Powder Extract	Anti-inflammatory Antioxidant Hepatoprotective Anticarcinogenic Anti-diabetic Antimicrobial Antidepressant Lowering cholesterol, triglyceride and low-density lipoprotein (LDL) levels	[58,59,60]
*Ganoderma lucidum* aggregate	Reishi	In China as Lingzhi or herb of spiritual potency	Ganodermataceae Or Polyporaceae	Mycelia Spores Fruit body	Polypore mushroom grows on decaying hardwood trees	Powders Dietary supplements Tea	Immunomodulation Anti-inflammatory, Antioxidant Radical-scavenging Analgesic, Chemo-preventive Antitumor, Chemo and radio protective, Anti-atherosclerotic, Sleep promoting Antimicrobial, Hypolipidemic, Anti-fibrotic Hepatoprotective, Anti-diabetic, Anti-androgenic Anti-angiogenic, Anti-aging, Estrogenic activity, Anti-ulcer	[61,62,63]
*Ganoderma tsugae* Murrill	Hemlock varnish shelf		Ganodermataceae Or Polyporaceae	Fruit body Mycelium	Flat polypore mushroom	Extract	High antioxidant Immunomodulatory, Antitumor, Anti-inflammatory Anti-fibrotic, Antiautoantibody formation	[64]
*Gentiana burseri* subsp. *villarsii* (Griseb.) Rouy (Syn. *G. macrophylla* Pall., *G. macrophylla* Bertol.)	Large leaf gentian	qin jiao in Chinese	Gentianaceae	Roots Radix Gentianae macrophyllae Flowers	A glabrous pale green perennial with erect, gross, 3–6 mm in diameter and 40–70 cm long stems and very large, acute, elongate-lanceolate and up to 40 cm long leaves with intensely bluish-violet and bell-shaped flowers and brown, bright and wingless seeds.	Extract	Anti-inflammatory, Wound healing, Analgesic, Antioxidant, Immunomodulation Hepatic protection, Joint protection, Cardio-protective Neuro-protective Anti-influenza	[65,66,67,68]
*Glycyrrhiza glabra* L.	Licorice Liquorice	“Sus”, “Mahak”,“Mehak”, “Bikh-e-Mahak”, in Persian ethnomedicine, “Shirin Bayan” (Persian) and “Irk-es-sus” (Arabic).	Fabaceae	Roots	An herbaceous perennial plant, growing to 1 m in height, with pinnate leaves about 7–15 cm long, with 9–17 leaflets. The flowers are 8–12 mm long, purple to pale whitish blue, produced in a loose inflorescence. The roots are stoloniferous.	Extract	Immunomodulatory Anti-inflammatory Antiapoptotic Neuroprotective Nephroprotective	[69,70,71,72]
*Linum usitatissimum* L.	Flax Flaxseed linseed	Cooler parts of Asia, Europe and Mediterranean regions In Persian as Bazr-e katan	Linaceae	Seeds	Annual small flowering plant with an approximate 60–100 cm length and 5 petal pale blue flowers and dark to yellow, oval, flat and pointed seed	Oil Extract Bread	Antioxidant, Immunomodulatory, Anti-inflammatory Antimicrobial, Antiprotozoal, Analgesic, Antihyperlipidemia, Antihyperglycemic, Antitumor	[73,74,75]
*Nelumbo nucifera* Gaertn. *(Syn.: Nelumbium* *nelumbo, N. speciosa, N. speciosum, Nymphaea nelumbo etc.)*	Lotus	sacred lotus, Laxmi lotus, Indian lotus, Egyptian bean In Chinese, seeds are called Lian Zi Xin, In India (lotus, kamala, pundarika or padma)	Nelumbonaceae	All parts of the plant (seeds, root, flowers, leaf, stem)	An aquatic species. The flowers (up to 30 cm in diameter) are on thick stems rising several centimeters above the leaves (that spread up to 80 cm in diameter). The leaf stalks grow up to 200 cm long while the peltate leaf blade or lamina can have a horizontal spread of 1 m	Extracts	Anti-inflammatory Immunomodulatory Antipyretic Antioxidant Cardioprotective Hepatoprotective	[76,77]
*Ophiocordyceps sinensis* (Berk.) G.H.Sung, J.M.Sung, Hywel-Jones and Spatafora (syn. *Cordyceps sinensis*)	Caterpillar fungus	In Tibet as yartsa gunbu In Chinese as “Dong Chong Xia Cao” or “winter worm summer grass” In India as keera jhar, keeda jadi, keeda ghas	Clavicipitaceae Ophiocordycipitaceae	fruiting body	A mushroom with plant-like fruiting body fill with mycelia	Oral liquids Capsules	Immunomodulatory Anti-inflammatory Antioxidative Anti-fibrogenic Antitumor Anti-thrombotic Anti-viral Antifungal	[78,79]
*Paeonia lactiflora* Pall.	Chinese peony Chinese herbaceous peony Common garden peony White Peony	East Asia In Chinese as “Bai Shao Yao”.	Paeoniaceae Or Ranunculaceae	Root (*Radix Paeoniae Alba)*	Herbaceous perennial flowering plant is about 60–100 cm tall with fleshy roots, annual stems and large compound leaves. The flower buds are large and round, opening into large flowers with 5–10 white, pink, or crimson petals and yellow stamen.	Dried root bark Extract	Anti-inflammatory Immunomodulatory Analgesic Antioxidative Hepatoprotective	[80]
*Paeonia × suffruticosa* Andrews	Cortex Moutan (king of flowers)	In China as *mǔdān* or Mudanpi	Ranunculaceae or Paeoniaceae	Roots bark	Collective name of cultivated tree peonies, originated from the hybridization of multiple species of wild tree peonies, belong to P. suffruticosa complex. A bush or a tree with attractive flowers and woody stems	Dried root bark Extract	Anti-inflammatory Antioxidative Anti-allergic Antitumor Cardiovascular System Protective Anti-Diabetic Neuroprotective Hepatoprotective Analgesic Sedative	[81]
*Rehmannia glutinosa* (Gaertn.) Liboschitz ex Fischer and C. A. Meyer	Rehmanniae Radix	In China as Di-Huang	Orobanchaceae or Scrophulariaceae	Roots Root tuber	A perennial plant that the roots of it are thick and yellowish in color, while the stems have grayish short hairs. The leaves appear to be gathered at the roots with edges that are blunt and serrated. The flowering form is raceme and blooms purple flowers from June to July.	Extract	Anti-inflammatory Antitumor Immunomodulatory Neuroprotective Hypoglycemic Cardioprotective Antioxidant Hematopoiesis promotion Antianxiety	[82,83]
*Scutellaria baicalensis* Georgi	Baikal skullcap Chinese skullcap Wogonin, baicalin, and baicalein	In China as *huángqín*	Lamiaceae	Roots and rhizomes	A perennial flowering herb with thick, fleshy, elongated and branched rhizome up to 2 cm in diameter. The leaves are lanceolate to linear lanceolate, 1.5–4.5 cm in length and 0.5–1.2 cm in width. The fruits are hard, ovoid, dark brown with a tumor, 1.5 mm in height, and 1 mm in diameter	Dried Extract	Anti-inflammatory, Antioxidant, Antitumor, Antibacterial, Antiviral, Liver protection, Effects on the nervous system, Effects on the immune system	[84,85,86]
*Tripterygium wilfordii* Hook. f.	-Thunder god vine -Thunder duke vine	In China (Mandarin) as léi gōng téng	Celastraceae	Roots without bark	wood vine plant perennial vien	Extract	Anti-inflammatory Immunomodulatory Immunosuppressive Anti-cancer Anti-fertility	[87,88]
*Urtica dioica* L.	Common nettle Burn nettle Stinging nettle	Europe Asia Africa	*Urticaceae*	leaves Roots Seeds Aerial parts	A perennial herbaceous plant with underground rhizome and bi-arch root and erect, green and quadrangular stem with dark-green, oblong or ovate leaves and small dioecious, brown to greenish flowers. Tinging trichomes cover both stems and leaves and contain a fluid.	Extract	Anti-inflammatory Antioxidant Analgesic	[89]

**Table 2:** Purified molecules from natural sources and their derivatives, fatty acids, minerals, and vitamins with reported efficacy against lupus (↓: decrease in activity or level; ↑: increase in activity or level).

Phytochemicals						
Compound Name	Class of Compound	Natural Source	Type of Study	Method	Result or Side Effect or Mechanism	Ref.
Antroquinonol	Farnesylated quinone	*Antrodia camphorata*	In vivo	NZB/NZW F1 mice were used Doses: 15 mg/kg antroquinonol orally for 5 weeks.	- ↓ hematuria, proteinuria and IL-18 production - Inhibited T cell proliferation and induced Treg cell suppression - ↓ reactive oxygen species and nitric oxide production - ↑ Nrf2 activation - Inhibited NF-ĸB activation	[93]
			In vivo	SLE-prone NZB/W F1 mice Doses: 100, 200, and 400 mg/kg of the mycelial extract of *A. camphorate* for 5 consecutive days per week for 12 weeks via gavage.	- ↓ proteinuria, creatinine and serum BUN levels - ↓ the thickness of the kidney glomerular basement membrane - ↓ the production of TNF-α and IL-1β	[46]
(*S*)-Armepavine	Benzylisoquinoline alkaloid	*N. nucifera*	In vivo	MRL/MpJ-lpr/lpr mice *Doses:* 5 and 10 mg/kg/day of oral (S)-armepavine, for 6 weeks.	- ↑ the life expectancy of mice - Inhibit splenocytes proliferation - prevent lymphadenopathy - Inhibit the expression of IL-2, IL-4, IL-10 and IFN-γ genes - Inhibit T cells proliferation - ↓ proteinuria and anti-dsDNA autoantibody	[94]
Artemisinin	sesquiterpene lactone	*A. annua*	Clinical trial	Treatment group: 31 patients were treated with 2–4 g/d of *C. sinensis* and 0.6 g/d of artemisinin. *C. sinensis* was taken before three main meals and artemisinin after three main meals for three years. Control group: 30 patients received tripterygiitotorum and/or Baoshenkang.	- The creatinine clearance rate did not change - The complement C3 level stabilized in the normal range - The combination of C. sinensis and artemisinin could be effective in the prevention of recurrence of lupus nephritis	[95]
			In vivo	MRL lpr/lpr mice aged 12 weeks Doses: 40 µg/kg/d of H1-A daily for 8 weeks.	- ↓ the production of anti-ds-DNA, lymphadenopathy, and proteinuria - Improved renal function - No significant changes have been observed in immune complex deposition	[96]
Beta-Aminoarteether maleate (SM934)	Synthetic artemisinin derivative (Sesquiterpenes)	an analogue of artemisinin	In vivo, in vitro and ex vivo	In vitro: the effects of SM934 on the activation of polyclonal CD4^+^ T cells and the differentiation of naive CD4^+^ T cells were investigated. In vivo: preventive or therapeutic effects of SM934 were investigated in MRL/lpr mice. Ex vivo: the treatment mechanisms were investigated.	- ↓ the production of IFNγ and IL-17 by polyclonal CD4+ T cells activated by T cell receptor rearrangement - Development of naive CD4+ T cells into Th1 and Th17 cells, but not Treg cells - Attenuate the renal lesion severity - ↓ proteinuria, ↓anti-dsDNA autoantibody - ↓ the spleen sizes - ↓ the levels of serum IFN-Y and blood urea nitrogen - ↑ lifespan of mice - Inhibit the production of Th1 and Th17 while an increase in the level of Treg cells - Inhibit the complete activation of STAT-1, STAT-3, and STAT-5 proteins	[97]
Baicalin	Flavone glycoside	*Scutellaria baicalensis*	In vivo	MRL/lpr lupus-prone mice Doses: 200 mg/kg of baicalin daily for 4 weeks, intraperitoneally	- ↓ anti-ds-DNA antibody - ↓ urine protein levels - Inhibit mTOR activation - ↓ reduce mTOR agonist-mediated Tfh cell expansion - ↑ Tfr cells - ↓ IL-21 production - Inhibit Tfh cell differentiation and Foxp3^+^ regulatory T cell differentiation	[98]
			In vivo	Adult female BALB/c mice Doses: 50 mg/kg of baicalin, orally, once in a day for 10 days.	- ↓ production of proinflammatory cytokines such as TNF-α, IL-6, IL-10, and IFN-γ - Inhibit the overproduction of IL-6 and PGE2 - Downregulate the aberrant activation of T cells	[99]
*Curcumin*	Diarylheptanoid	*C. longa* (turmeric)	Clinical trial	The study was conducted on six SLE patients and six healthy individuals. CD4^+^ of these people was collected then stimulated by Th17 differentiating factors and exposed to 0.1 and 1 µg/mL curcumin.	- ↓ Th17 percentage - ↓ IL-17a productions - ↑ Treg percentage - ↑ TGF-β1 productions - Modulate Th17/Treg balance	[100]
			In vivo and in vitro	Female MRL/lpr mice were treated with 200 mg/kg of curcumin for 8 weeks.	- ↓ proteinuria, ↓ renal inflammation and, ↓ spleen size - ↓ NLRP3 inflammasome activation - Inhibit anti-dsDNA serum induced expression of NLRP3 inflammasome in podocytes	[101]
			In vivo	Female NZBWF1 have been treated with 500 mg/kg/day curcumin by oral gavage for 14 days.	- Weight and body composition were maintained - ↓ spleen weight and renal injury (glomerulosclerosis)	[102]
			In vivo	Female BALB/c mice with lupus (0.5 cc pristane, i.p.) Doses: 0, 12.5, 50, and 200 mg/kg bw/day curcumin intragastrically daily for 16 weeks.	- ↓ arthritis score and proteinuria level - No significant alteration in body weights - ↓ Th1, Th2, and Th17 percentages - ↑ Treg percentages - ↓ serum IL-6 and IFN-α levels - ↓ the level of antinuclear antibody	[103]
Dihydroartemisinin	A derivative of artemisinin (Sesquiterpenes)	*A. annua*	In vivo	Male BXSB mice Doses: of 5 mg/kg, 25 mg/kg, or 125 mg/kg	- ↓ TNF-alpha production - ↓ NF-κB activation - ↓ p65 subunit expression - Inhibit NF-κB translocation to the nucleus - Inhibit IκB-α protein degradation	[104]
			In vivo	BALB/C female mice were infected with *Toxoplasma gondii* or *Plasmodium berghei*. Doses: 2 mg of dihydroartemisinin daily. Spleen and blood samples were collected.	- ↓ the number of B cells in the bloodstream and spleen cells - ↑ the ratio of T helper to CD8^+^ T cells - ↓ the proinflammatory cytokines	[105]
			In vivo and In vitro	Female BALB/c mice have been used. Dose:100 mg/kg of dihydroartemisinin, the fourth group was given 5 mg/kg prednisolone, and the last group was given a combination of prednisolone and dihydroartemisinin with the same previous doses. Dihydroartemisinin and prednisolone were administered daily and orally for two months.	- Restore the Treg/Th17 balance - Inhibit inflammation - ↑ the levels of TGF-β, IL-17 and Foxp3 - ↓ transcription of RORγt - Inhibit Th17 cell differentiation - Induce Treg cell differentiation	[106]
Emodin	Anthraquinone	*Rheum palmatum*	In vivo	BXSB lupus mice Mice were treated with different doses of emodin for 30 days.	- ↓ proteinuria - ↓ the expression of ICAM 1 in the renal glomerulus	[107]
			In vivo	Lupus-prone male BXSB mice Doses: 0, 5, 10, and 20 mg/kg/day emodin for 30 days	- ↓ glomerular levels of TNF-α, ICAM-1 - ↓ fibronectin - ↓ the levels of urinary protein and serum anti-dsDNA antibody	[108]
Esculetin	Coumarin	*C. intybus* and *H. paniculate*	In vivo and In vitro	MRL/lpr mice Doses: esculetin (20 mg/kg and 40 mg/kg) from 10 to 20 weeks	- Attenuated renal impairment by reducing - ↓ BUN, serum creatinine and albuminuria - Ameliorated the glomerular hypertrophy, tubular interstitial fibrosis and mononuclear cell infiltration into interstitium. - Down-regulated complement cascade, inflammation and fibrosis pathway - Up-regulated Nrf2-related antioxidation genes - Inhibited the complement activation (both classical and alternative pathways) - Blocked the C3 convertase (C4b2a)	[109]
*Glycyrrhizin (Glycyrrhizic acid)*	Triterpenoid saponin	*G. glabra*	In vivo and In vitro	Female BALB/c mice received 0.5 mg/day of glycyrrhizin for two months.	- Inhibited HMGB1 function - Caused sharp decline in serum HMGB1 levels - reduced the severity of SLE	[110]
			In vivo and In vitro	Glycyrrhizin (10 mg/kg) was injected every other three days into female BALB/c mice for 12 weeks.	- ↓ levels of anti-dsDNA antibodies - ↓ levels of inflammatory cytokines - ↓ glomerular IgG and C3 deposition - ↓ proteinuria	[111]
			In vitro		- nhibit the immunocomplex formation of 60S acidic ribosomal P proteins from porcine liver	[112]
Mangiferin	Xanthone	*A. aspheloidis*	In vivo and In vitro	lupus-prone B6/gld mice Doses: 20 or 40 mg/kg/day of mangiferin for 12 weeks, orally	- Suppressed mTOR signaling pathways - Upregulated CD4^+^ FoxP3^+^ Tregs - Inhibited T cell proliferation - Improved renal immunopathology - ↓ renal T cell infiltration - ↓ serum creatinine - ↓ urinary protein levels - ↑ CD4^+^ FoxP3^+^ Treg frequencies in the spleens, lymph nodes, and kidneys	[113]
Salvianolic acid A	A type of phenolic acid	*Salvia miltiorrhiza*	In vivo	BALB/c mice Doses: 5 mg/kg/day of salvianolic acid A for 5 months	- ↓ anti-Sm autoantibodies - Inhibit phosphorylation of IKK, IκB and NFκB in kidney tissue - ↓ pathological effects	[114]
Total glucosides of paeony (TGP)	Glucosides of paeony	*P. lactiflora*	Clinical trial	One group of 29 cases received TGP for 5 years or more, the other group of 47 cases received TGP for 1 year or more (but less than 5 years), and the third group was the control.	- Decreased the required daily dose of prednisolone and cyclophosphamide - ↓ SLEDAI score - No significant difference has been observed in urinary protein - No side effects were observed following TGP use	[115]
			Clinical trial	70 SLE patients were divided into control and treatment groups. Both groups used conventional medicine, but the treatment group also received TGP for three months.	- Decreased dose of glucocorticoids required - A combination of TGP and glucocorticoids was suggested to be beneficial	[116]
			Meta-analysis on clinical trials	- Total of 23 articles were included - 792 patients overall in the treatment group and 781 patients overall in the control group	- ↓ IL-18 and IL-6 levels - ↓ anti-dsDNA level - ↓ serum creatinine levels - ↑ Albumin and complement C3, C4 levels - ↓ the disease activity of SLE - ↓ the incidence of adverse reactions - was more effective and safer when used in combination with conventional therapeutics - improved immunoglobulins, (IgA, IgM and, IgG), ESR, CRP, 24 h urine protein, and recurrence rate	[117]
			In vivo	MRL/lpr mice were treated with 50 mg/kg/d of TGP by gavage for 4 weeks.	- ↓ ERα expression - ↑ DNMTs expression - ↓ renal damage - ↓ expression of IFN-γ, IL6 and IL12 cytokines - ↓ anti-dsDNA autoantibody	[118]
			In vivo	The effect of TGP on lupus nephritis has been investigated on MRL/lpr mice	- ↓ the urinary protein contents - ↓ the level of anti-dsDNA antibodies and ANA	[119]
			In vitro	CD4^+^ T cells were treated with doses of 0, 62.5, 312.5, and 1562.5 mg/L of TGP for 48 h to elevate the effect of TGP on expression and DNA methylation status of ITGAL gene (CD11a) in CD4^+^ T cells.	- Down-regulate the level of ITGAL mRNA and protein - ↑ DNA methylation of the ITGAL promoter - ↓ CD11a gene expression	[120]
Triptolide	Diterpenoid epoxide	*T. wilfordii*	In vitro	To evaluate the effect of triptolide doses: 0, 5, 10, 30 μg/L of triptolide	- Inhibited the differentiation and maturation of DCs. - ↓ the immune function of DCs. - ↓ secretion of IFN-α, IL-6, and TNF-α	[121]
			In vivo	BALB/c-in nude mice Dose: 5 mg/kg/d of triptolide, orally blood samples were collected before treatment and 1, 3, and 6 months after treatment.	- ↓ the percentage of CD8^+^, Tcl, Thl cells, CD4^+^/CD8^+^, Thl/Th2 and Tcl/Tc2 - ↑ the percentage of CD4^+^, Tc2 and Th2 cells	[122]
			In vivo	(NZB×NZW) F1 mice Dose: 6 µg of triptolide or tripdiolide, orally for 15 weeks.	- ↓ levels of BUN, proteinuria, and anti-dsDNA antibody - ↓ production of cytokines such as IL-6 and TNF and monocyte chemoattractant protein 1	[123]
			In vivo	Female MRL/lpr mice Control group received 20 mg/kg/w of cyclophosphamide. treatment groups received 0.2 or 0.3 mg/kg/d of triptolide for 13 weeks.	- ↓ levels of proteinuria, serum anti-dsDNA - ↓ renal histopathologic assessment - ↑ the proportion of Treg - Induce the expression of the miR-125a-5p	[124]
(5R)-5-Hydroxytriptolide (LLDT-8)	Diterpenoid epoxide		In vivo	The effect of LLDT-8 on lupus nephritis was investigated so, female MRL/lpr mice were treated with 0.125 mg/kg/2 days of LLDT-8 for 9 weeks.	- ↓ proteinuria - ↓ serum creatinine - ↓ glomerular IgG deposits - ↓ histopathology - ↑ the lifespan of mice - ↓ the expression of inflammatory cytokines such as IFN-γ, IL-17, IL-6, TNF-α - Inhibit immune cell infiltration in the kidneys	[125]
Fatty acids, vitamins, and minerals						
Fatty acids	Fatty acids		*meta-analysis*	A systematic review and meta-analysis study has been conducted to evaluate the effect of omega-3 fatty acids on SLE disease activity in adults.	- Omega-3 fatty acids could provide therapeutic benefit in addition to immunosuppressive regimens used for SLE - Omega-3 fatty acids: ↑lifespan and ↓autoantibody levels	[22]
			In vivo	Weanling (NZB 3 NZW) F1 female mice mice were switched to semi purified diets containing 10% corn oil as control oil and FOs enriched in either EPA or DHA	- Omega-3 fatty acids: - regulate blood pressure and proteinuria and ↓anti-dsDNA levels and ↓ TNF-α, IL-1α, IL-1β and IL-2 - DHA: - Inhibit IL-18 induction and ↑lifespan and suppress glomerulonephritis	[126]
Vit A	Vitamin		*Clinical trial*	45 female patients with SLE and 45 healthy age-matched and sex-matched patients as control group	- Regulate the balance between Th17 and Treg	[127]
			*Clinical trial*	Sixty-two female SLE patients and sixty-two female controls	- ↓ level of Th17 - ↑ level of Treg	[128]
Vitamin B	Vitamin		*Clinical trial*	The association of each nutrient intake with the risk of developing active disease was investigated in 216 patients who initially had inactive disease. The association with atherosclerotic vascular events was assessed in 196 women with inactive disease and no history of atherosclerotic disease at baseline.	- Vitamins B6, B12, and folate: ↓homocysteine levels and improve atherosclerosis and ↓ levels of inflammatory cytokines and CRP - Vitamin B6: ↓homocysteine and ↓ risk of active disease	[129]
			*Clinical trial*	Seventeen patients with SLE were used for 12 weeks	- ↓ sodium intake and maintain adequate intakes of most nutrients except B12, dietary fiber, iron, calcium, and folate. - Niacin: ↓ triglyceride and LDL-C levels	[130]
Vitamin C	Vitamin		*Clinical trial*	279 female patients with SLE were followed over 4 years	- dietary nutrients may modify clinical course of disease in female patients with SLE. - Vitamin C intake is inversely associated with the risk of active disease. - Vitamin C intake may prevent the occurrence of active SLE disease.	[131]
Vitamin D	Vitamin		*Clinical trial*	25-OH vitamin D levels were measured in 198 consecutively recruited SLE patients.	- Inhibit dendritic cell activation and maturation	[132]
			*Systematic review*	1268 articles have been reviewed to determine whether supplementation with vitamin D can reduce the risk or modify the course of autoimmune diseases.	- Basic, genetic, and epidemiological studies indicate a potential role of vitamin D in the prevention of autoimmune diseases.	[133]
Vitamin E	Vitamin		*Clinical trial*	12 women among 36 outpatients received vitamin E (150 to 300 mg/day) together with prednisolone (PSL).	- ↓ the generation of autoantibodies - vitamin E can suppress autoantibody production via a mechanism independent of antioxidant activity.	[134]
Iron	Mineral		In vivo	Weanling female MRL/MPJ-lpr/lpr mice were used. Mice were fed diets with 3, 10, 35, and 250 mg Fe/kg diet	- ↑ cell damage and renal lesions - Worsen renal impairment	[135]
Selenium	Mineral		In vivo	NZB/NZW female mice Selenium supplementation was provided by adding sodium selenite to the drinking water at 0, 2, or 4 parts per million (mg/L).	- ↑ survival - ↑ levels of natural killer cell activity	[136]
			In vivo and in vitro	They have investigated the impact of Se on B cells and macrophages using in vitro Se supplementation assays and the B6.Sle1b mouse model of lupus with an oral Se or placebo supplementation regimen.	- Inhibit the activation, differentiation, and maturation of macrophages and B cells - ↓ splenomegaly and splenic cellularity compared	[137]

**Table 3:** Medicinal plants, mushrooms, and fungi with reported efficacy against lupus.

Plant Name	Other Names	Type of Study	Method	Result or Side Effect or Mechanism	Ref.
*Antrodia camphorata*	Stout camphor fungus	In vivo	SLE-prone NZB/W F1 mice took 100, 200, and 400 mg/kg of *A. camphorata* extract, orally on 5 consecutive days per week for 12 weeks.	- ↓ urine protein - ↓creatinine - ↓ serum BUN levels - ↓ the kidney glomerular basement membrane’s thickness	[46]
*Artemisia annua* Pall.	Annual wormwood, sweet wormwood, Chinese wormwood, sweet sagewort sweet Annie	In vivo and in vitro	Six groups of female ICR mice were used and there were 5 mice in each group. Splenocytes of immunized mice were isolated and exposed to different concentrations, and finally the number of specific antibodies was counted by indirect ELISA.	- ↓ the levels of a series of antibodies	[228]
*Astragalus propinquus Schischkin (syn. Astragalus membranaceus* (Fisch.) Bunge)	Mongolian milkvetch	In vitro	NK activity of peripheral blood mononuclear cells (PBMC) from 28 patients with SLE was measured using enzyme-release assay.	- ↓ NK activity - ↓ disease activity	[229]
*Bryophyllum pinnatum* (Lam.) *Oken*	Kalanchoe pinnata, Zakham-e-hyat, life plant, cathedral bells	In vivo and in vitro	BALB/c mice were treated with different doses of ethanolic extract of *B. pinnatum* leaves Doses: 10.5, 21, and 42 mg/kg/day for 12 weeks.	- ↓ TNF-α, IL-17, IL-12, CRP, and matured B cells - ↑ complement C3 and C4 and TGF-β - No specific side effects have been reported	[230,231]
		In vitro	The effect of ethanolic extract of *B. pinnatum* leaves on spleen cells of BALB/c mice Doses: 0, 0.02, 0.1, or 0.5 µg/mL	- ↓ B cells maturation - ↑ B cells apoptosis - ↓ NF-ĸB p65 expression	[232]
		In vivo and in silico	The effect of aqueous extract of *B. pinnatum* leaf on lupus nephritis in female Balb/c mice Doses: 200, 400, or 600 mg/kg/day, orally for 21 days	- ↓ proteinuria levels - ↓ glomerular inflammation - bryophyllin A is probably the active compound of *B. pinnatum* for its anti-inflammatory effect	[233]
*Camellia sinensis* (L.) Kuntze	Tea	Clinical trial	68 SLE patients were divided into control and study groups. The study group was treated with 1000 mg of *C. sinensis* extract daily for 12 weeks.	- ↑ the quality of life of patients - ↑ general health - ↓ disease activity	[234]
*Curcuma longa* L.	Turmeric	Clinical trial	24 patients with lupus nephritis divided into study and control groups. The study group was treated with 1500 mg of turmeric daily for 3 months.	- ↓ proteinuria and hematuria - ↓ systolic blood pressure - No side effects have been observed	[235]
*Ganoderma lucidum and Ganoderma tsugae*	Lingzhi Reishi	In vivo	Female MRL/lpr mice with mild, moderate, and severe lupus Doses: Initially, 500 mg/kg/day was administered orally for 7 days, and then 50 mg/kg/day was injected intraperitoneally for 7 days.	- Significant reduction in anti-ds-DNA - ↑ the percentages of IL-10, CD4^+^, CD25^+^, Foxp3^+^ and Treg cells - ↑ the concentrations of IL-2 and IL-12P70 - ↓ the concentrations of IL-21, IL-10, and IL-17A	[236]
		In vivo	All groups of NZB/NZW F1 mice (two months of age) were given standard laboratory chow feeding. The first study group was given 0.1 cm^3^ of oral ganoderma extract, and the second study group was given 0.2 cm^3^ of oral ganoderma extract, daily. The third study group was given 0.5 mg/kg/day of prednisolone.	- ↑ life expectancy - ↓ anti-dsDNA autoantibody, proteinuria, parenchyma and perivascular mononuclear cell infiltration	[237]
*Gentiana macrophylla* Pall.	Qinjiao	Clinical trial	Treatment group: 62 patients with SLE were treated with *G. macrophylla* complex tablets (10 tablets BID or 5 tablets TID) with 10 to 30 mg of prednisolone daily. Control group: 19 SLE patients were treated with prednisolone alone.	- The recovery rate was significantly higher in the treated group - Improvement of nephropathy, erythema, arthralgia and restoration of ESR, LE cells, C3 and CH50 - No significant complication was observed	[238]
		In vivo and In vitro	Female NZB/W F1 mice divided into the control group, the cholesterol consuming group and the cholesterol and *G. macrophylla* consuming group. (a 12-week study, heart tissues were examined)	- ↓ cholesterol-aggravated apoptosis - ↑ IGF-1 survival signal - ↑ anti-apoptotic proteins	[67]
		In vivo and In vitro	Female NZB/W F1 mice—an 8-week study	- reduced liver inflammation	[239]
*Linum usitatissimum* L.	Flaxseed, linseed	Clinical trial	23 patients with lupus nephritis (15 volunteers remained) Doses: 30 g/day A two-year nonplacebo-controlled crossover study	- Viscosity of serum and plasma lipids remained unchanged. - ↓ serum creatinine - ↓ microalbumin	[240]
		Clinical trial	8 patients with lupus nephritis Doses: 15, 30, and 45 mg of flaxseed daily for four weeks, with a washout of 5 weeks between doses.	- ↓ blood viscosity and LDL - Inhibit AF-induced platelet aggregation - ↓ serum creatinine and proteinuria - ↑ complement C3 - ↓ expression of CD11b	[241]
*Ophiocordyceps sinensis* (syn. *Cordyceps sinensis*)	Yartsa gumba caterpillar fungus	meta-analysis study	This meta-analysis study was conducted on a total of 14 studies comprising 1301 participants.	- Consumption of *O. sinensis* mycelium for lupus nephritis is more effective than not using it. - There was no significant difference between the Bailing group and the control group in anti-ds-DNAIgM levels and complement C3 levels. - Improvement of other indicators of the disease, such as SLEDAI score, Alb, 24 h urinary protein, serum creatinine, and the number of effective treatments and complications.	[142]
		In vivo	Lupus-prone (NZB/NZW) F1 hybrid mice with different ages (three, six, and eight months) Doses: 2.4 mg/g/day of cultured mycelia of *C. sinensis* orally	- In groups who started taking it at the ages of 3 and 6 months, survival increased, proteinuria decreased, and titers of anti-double-stranded DNA antibody decreased. - ↓ the percentage of CD4^+^ T cells in PBMC and ↑ the percentage of CD8^+^ T cells - Early administration of *C. sinensis* reduces the severity of lupus disease	[242]
		In vivo	MRL lpr/lpr mice aged 12 weeks Doses: 40 µg/kg/d of H1-A daily for 8 weeks.	- ↓ the production of anti-ds-DNA, lymphadenopathy, and proteinuria - Improved renal function - No significant changes have been observed in immune complex deposition.	[96]
		In vivo	The plants studied in this article include: *Cordyceps sinensis*, *Anqelica sinensis*, *Atractylodes ovata*, *Codonopsis pilosula*, *Ligustrum lucidum*, and *Homo sapiens*. 144 NZB/NZW F1 mice at one month of age were divided into 19 groups	- *C. sinensis* was found to inhibit anti-ds-DNA production and increase the lifespan of mice. - *A. sinensis* does not inhibit anti-ds-DNA production, but it has been able to increase the lifespan of mice.	[243]
*Paeonia lactiflora Pall.*	Chinese peony common garden peony shaoyao	In vivo and In vitro	MRL/lpr lupus mice Received Radix Paeoniae Rubra for 12 weeks	- ↓renal pathological damage - ↓urinary protein levels - ↓ the expression of ICAM-1, VCAM-1 and PECAM-1	[244]
*Paeonia × suffruticosa* Andrews	mǔdān Moutan Cortex	Clinical trial	Moutan Cortex extract group: 84 patients control group: 84 patients	- ↓ the percentage of Th17 cell, - ↑ the percentage of Th1 cell - ↓ IL-6 level, -↓ ESR and SLEDAI score - ↓ side effects	[245]
*Rehmannia glutinosa* (Gaertn.) DC.	*Rehmanniae Radix* (Di Huang)	Clinical trial	72 patients were divided into control and treated groups. The control group was given prednisolone and cyclophosphamide. In the treatment group *Radix Rehmanniae* and *Radix Astragali* was added to the treatment regime for 6 months.	- The dose reduction of prednisolone was greater in the treated group. - Patients who had to increase the dose of prednisolone due to aggravating disease were fewer in the treated group. - Infection, cardiovascular anomalies, hot flush, insomnia, and Cushing’s syndrome were less common in the treated group. - There was no difference in blood immunoglobulin G and blood complement 3 in the two groups. - The protein in the 24 h urine was lower in the treated group.	[246]
		In vitro	The effect of fresh Rehmanniae radix methanol extract has been investigated in adult female BALB/c mice. Mouse splenocytes were investigated.	- ↓ inflammatory cytokines such as IL-2, IFN-γ, IL-6 and IL-10	[247]
*Scutellaria baicalensis Georgi*	Baikal skullcap Chinese skullcap	In vitro	In vitro study on splenocytes of pristane-induced lupus BALB/c mice.	- Downregulated the production of proinflammatory cytokines such as TNF-α, IL-6, IL-10 and IFN-γ. - The expression of CD69^+^ CD4^+^ T cells and CD4^+^ T cells decreased but CD8^+^ did not decrease.	[248]
*Tripterygium wilfordii* Hook. F.	Thunder duke vine Thunder god vine Léi gōng téng	Clinical trial	There were 23 cases of lupus erythematosus, of which 15 had SLE and 8 had DLE. They were given 45 mg/day of crude extract of *T. wilfordii*. The control group consisted of 19 cases of SLE treated with prednisolone.	- The rate of improvement was almost the same in the two groups, and no significant difference was observed. - Recovery from erythematosus rash and arthralgia following use of *T. wilfordii.*	[249]
		Clinical trial	26 DLE cases treated with *T. wilfordii*	- Progress with varying degrees was observed in 24 cases.	[250]
*Urtica dioica* L.	stinging nettle grande ortie anonhasquara	Case report	One female lupus with status renal allograft and elevated serum creatinine was consuming immunosuppressants. She consumed an herbal remedy, consisting of a combination of *Agropyron repens* rhizome and *U. dioica* seed extracts (1:3, 5 mL, three times a day for 46 days), and then she received *U. dioica* seed extract as a monotherapy for three months.	- ↓ serum creatinine after consuming mixed herbal remedy - Serum creatinine levels were normalized to acceptable levels after monotherapy of U. *dioica* seed extract for three months.	[251]
		In vivo	MRL lpr/lpr mice Dose: 100 µg, inj., every 2 weeks for 4.5 months, blood samples were every 3	- Inhibited the development of overt clinical signs of lupus and nephritis. - Pathogenic T cell clones are thus found among the V8.3+ T cell population, which also contains an enlarged T cell clone. - Affected autoantibody production in a sex-dependent manner.	[252]

## Data Availability

Not applicable.
